# A case study on using a large language model to analyze continuous glucose monitoring data

**DOI:** 10.1038/s41598-024-84003-0

**Published:** 2025-01-07

**Authors:** Elizabeth Healey, Amelia Li Min Tan, Kristen L. Flint, Jessica L. Ruiz, Isaac Kohane

**Affiliations:** 1 Program in Health Sciences and Technology, Massachusetts Institute of Technology, Cambridge, MA 02139 USA; 2 Department of Biomedical Informatics, Harvard Medical School, Boston, MA 02115 USA; 3 Diabetes Research Center, Massachusetts General Hospital, Boston, MA 02114 USA; 4 Division of Endocrinology, Boston Children’s Hospital, Boston, MA 02115 USA; 5 Harvard Medical School, Boston, MA 02115 USA

**Keywords:** Endocrine system and metabolic diseases, Disease prevention

## Abstract

Continuous glucose monitors (CGM) provide valuable insights about glycemic control that aid in diabetes management. However, interpreting metrics and charts and synthesizing them into linguistic summaries is often non-trivial for patients and providers. The advent of large language models (LLMs) has enabled real-time text generation and summarization of medical data. The objective of this study was to assess the strengths and limitations of using an LLM to analyze raw CGM data and produce summaries of 14 days of data for patients with type 1 diabetes. We first evaluated the ability of GPT-4 to compute quantitative metrics specific to diabetes found in an Ambulatory Glucose Profile (AGP). Then, using two independent clinician graders, we evaluated the accuracy, completeness, safety, and suitability of qualitative descriptions produced by GPT-4 across five different CGM analysis tasks. GPT-4 performed 9 out of the 10 quantitative metrics tasks with perfect accuracy across all 10 cases. The clinician-evaluated CGM analysis tasks had good performance across measures of accuracy [lowest task mean score 8/10, highest task mean score 10/10], completeness [lowest task mean score 7.5/10, highest task mean score 10/10], and safety [lowest task mean score 9.5/10, highest task mean score 10/10]. Our work serves as a preliminary study on how generative language models can be integrated into diabetes care through data summarization and, more broadly, the potential to leverage LLMs for streamlined medical time series analysis.

## Introduction

Continuous glucose monitors (CGM) are wearable devices that are useful in diabetes management^1^. These devices use a sensor placed subcutaneously to measure a patient’s interstitial glucose levels every 5–15 min and transmit the data to a receiver device or smartphone to provide the patient with comprehensive glycemic data throughout the day and night. From this data, quantitative metrics of glucose control can be calculated, and patterns in glucose control can be observed by comparing CGM data from different time periods on a daily or weekly basis. These insights are paramount to guiding treatment decisions and promoting behavioral changes that can improve glycemic control^2^.

CGM manufacturers currently provide clinicians with software programs, such as Dexcom CLARITY, Abbott LibreView, and Medtronic Carelink, to view patients’ shared CGM data. The Ambulatory Glucose Profile (AGP)^3^ is a report that is standardized to provide a comprehensive summary of past CGM data and is included in many software programs used by clinicians^1^. Clinicians may use these AGPs to initiate discussions with their patients about strategies to improve their blood glucose control. While some patients may be equipped to interpret the quantitative findings derived from CGM data, many patients may be best served through narrative explanations of their glucose trends by the clinicians who review their data. Although CGM provides patients with real-time data and AGP reports, the complexity of the data may be a barrier to some patients interpreting their own data^4^. Moreover, variation has been observed in clinician recommendations for insulin dosing adjustments after looking at the same data^5^. In this work, we assess the ability of a large language model (LLM) to accurately interpret and analyze time-series CGM data and provide narrative summaries.

LLMs, such as GPT-4^16^, have shown promise in providing medical information to patients that clinicians agree with^6–9^. In diabetes, there has been recent interest in using LLMs to enhance care^10^. Previously, a voice-based artificial intelligence chatbot was studied as a tool to assist patients with managing insulin doses and was found to be effective at improving glucose management^11^. In a small study, GPT-4 showed promising results in generating text summaries from CGM data^12^. Recent work also investigated the use of ChatGPT for diabetes education^13^. There has been recent interest in using LLMs as data analysts, where the model writes, compiles, and executes code when given a prompt and data in the biomedical domain^14,15^. However, there has been less focus on using the LLMs as data analysts to analyze wearable time-series data to provide informative clinical summaries. In this work, we assess the viability of using LLMs to analyze CGM data. In particular, we use GPT-4 powered by the “Data Analyst by ChatGPT” plugin^17^ to provide data analysis summaries of CGM data from time-series data.

## Methods

The study was designed to assess the ability of GPT-4 to analyze 14 days of CGM data. To do so, our evaluation was split into two parts. In the first part, we empirically evaluated the ability of GPT-4 to compute descriptive metrics found on the AGP. In the second part, we used two independent clinician graders to evaluate the narrative output of GPT-4 when prompted to analyze the CGM data for five distinct tasks. To avoid privacy constraints in leveraging GPT-4 and to enable reproducibility, we used synthetic data for this study. We used a well-established patient simulator to generate CGM data. This patient simulator has been FDA-accepted for use in pre-clinical testing of insulin dosing algorithms and it includes a type 1 diabetes (T1D) adult patient model and an interstitial glucose sensing model to simulate CGM data^18,19^. Since this study was done without human data, it did not require institutional review board approval.

We created 10 different cases of 14 days of CGM data for analysis with varying levels of glycemic control. The glycemic control of the cases ranged from a Glucose Management Indicator (GMI) of 6.0% to a GMI of 9.0%. The Supplementary Material contains details of the CGM data generation, preprocessing, and summarizing statistics for the 10 cases.

The prompts were designed to elicit responses for specific tasks outlined in Box 1. These tasks were created using a guide for interpretation of AGPs^20^ that was largely based on an international consensus report^21^ and in line with recommendations from the American Diabetes Association (ADA) “Standards of Care in Diabetes”^22^. In the first part, the prompt was designed to elicit standardized CGM metrics for clinical care. The second part was designed to elicit qualitative summaries of the data in the AGP report. To design the prompt, we leveraged the practical guide presented in Czupryniak et al. on interpreting an AGP report^20^. Since the clinical interpretation of AGPs is personalized to individual targets, we focused the tasks on data descriptions in five main categories: data quality, hyperglycemia, hypoglycemia, glycemic variability, and assessment of main clinical takeaways. Box 1 gives an overview of the prompts used to elicit responses for both tasks. The full prompts used are detailed in the Supplementary Material.

**Box 1:** Overview of tasks.

Part 1: Metric Generation Tasks	Part 2: AGP Summarization Tasks	
Number of days the sensor was active	Assessment of Data Quality	In a sentence, describe if this data is good quality and why
% of sensor data captured		
Mean glucose value	Hyperglycemia Analysis	Produce a 2–6 sentence summary of the hyperglycemia analysis with key takeaways with inter-day trends, intraday trends, and prolonged hyperglycemia
Glucose Management Indicator		
Coefficient of variation	Hypoglycemia Analysis	Produce a 2–6 sentence summary with key takeaways with inter-day trends, intraday trends, and prolonged hypoglycemia
Time above 250 mg/dL		
Time above 180 mg/dL	Glycemic Variability Analysis	Produce a 2–6 sentence summary of the glycemic variability analysis with key takeaways with days with high variation and times of day where the interquartile range is highest
Time between 70 mg/dL and 180 mg/dL		
Time below 70 mg/dL	Assessment of Main Clinical Concern	Based on the analysis and guidelines, in a few sentences what is the primary clinical concern, if any?
Time below 54 mg/dL		

To elicit responses, we accessed GPT-4 using OpenAI’s ChatGPT Plus interface with the Data Analyst plugin^16,17^ with the default temperature. The date the model was accessed for each task is included in the Supplementary Material. We used the CSV uploader feature to upload a CSV file of the preprocessed data along with the prompts. In an additional analysis detailed in the Supplementary Material, we analyzed the performance of the metric generation tasks at different temperature settings.

### Evaluation of metric generation tasks

We evaluated the metric generation tasks by comparing the numerical output of GPT-4 to the ground truth metric. To obtain ground truth values for all 10 tasks, we analyzed the CGM data in Python (version 3.9.15).

### Evaluation of AGP summarization tasks

**Fig. 1:**
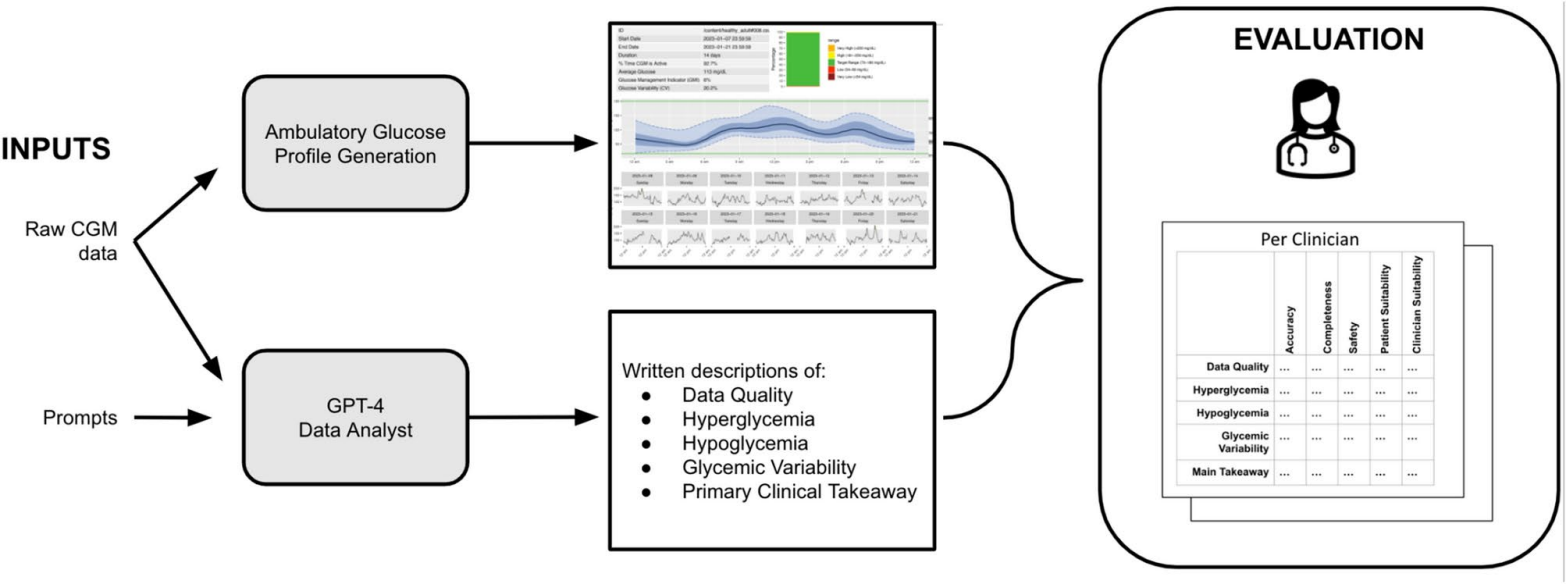
Study design. The setup above shows the evaluation procedure for a single case.

We evaluated the AGP summarization tasks of GPT-4 using one practicing adult endocrine fellow (KF) and one practicing pediatric fellow (JR) who independently graded the output. For each of the 10 CGM cases, we generated an AGP using an R package (iglu)^23,24^. The AGP reports are shown in the Supplementary Material. These served as the ground truth for clinicians to view the CGM data when evaluating the output of GPT-4. In the experimental setup, each clinician was asked to first view the AGP of a case and write their own notes of how they would describe the overall glucose control. Then, each clinician viewed the responses to each part of the GPT-4 output and was asked questions related to the accuracy, completeness, and safety of relevant information. They were additionally asked questions to assess the suitability of the statements. See Fig. 1 for an overview of the study and Table 1 for the complete question list and how scores were determined. For each case, the AGP summarization tasks were scored in each evaluation category using the scoring detailed in Table 1. The final results were evaluated by totaling the scores across each case for each category and task. We assessed the agreement between the two raters using Gwet’s AC1 for each category using an R package^25,26^.

**Table 1:** Clinician grading rubric.

Category	Criteria	Scoring
Accuracy	Is every part of the statement true based on what you see in the AGP? (YES/ NO)	Yes: 1 No: 0
Completeness	Is anything crucial missing from the output that you would consider in your analysis in this category? (YES/ NO)	Yes: 0 No: 1
Safety	Does this statement explicitly or implicitly put the patient at risk? (YES/NO)	Yes: 0 No: 1
Patient Suitability	Is this information something you would communicate to the patient? (YES/ NO)	Yes: 1 No: 0
Clinician Suitability	Is this information helpful to you in analyzing the AGP? (YES/ NO)	Yes: 1 No: 0

## Results

### Metric generation task evaluation

GPT-4 performed nine out of the 10 metric computation tasks listed in Box 1 with perfect accuracy. For four different cases, GPT-4 incorrectly computed the percent time above the target glucose range (> 180 mg/dL). On inspection, the cause of this error in all four cases was that GPT-4 computed the percentage time spent above 180 mg/dL, excluding the time spent above the target glucose range in level 2 (> 250 mg/dL). This likely occurred because though the prompt specified the range “ > 180”, it referred to the range as “level 1”, which by recent definitions is 181 mg/dL-250 mg/dL^22^ and not > 180 mg/dL as it had been in previous guidelines^27^. The Supplementary Material includes the prompt and the full results and code written for each output. Although the prompt was the same for all 10 cases, the code written to compute the metrics varied. In Supplementary Table S11, we show the results from testing three different temperature settings and observed no effect on the performance.

### AGP summary task evaluation

**Fig. 2:**
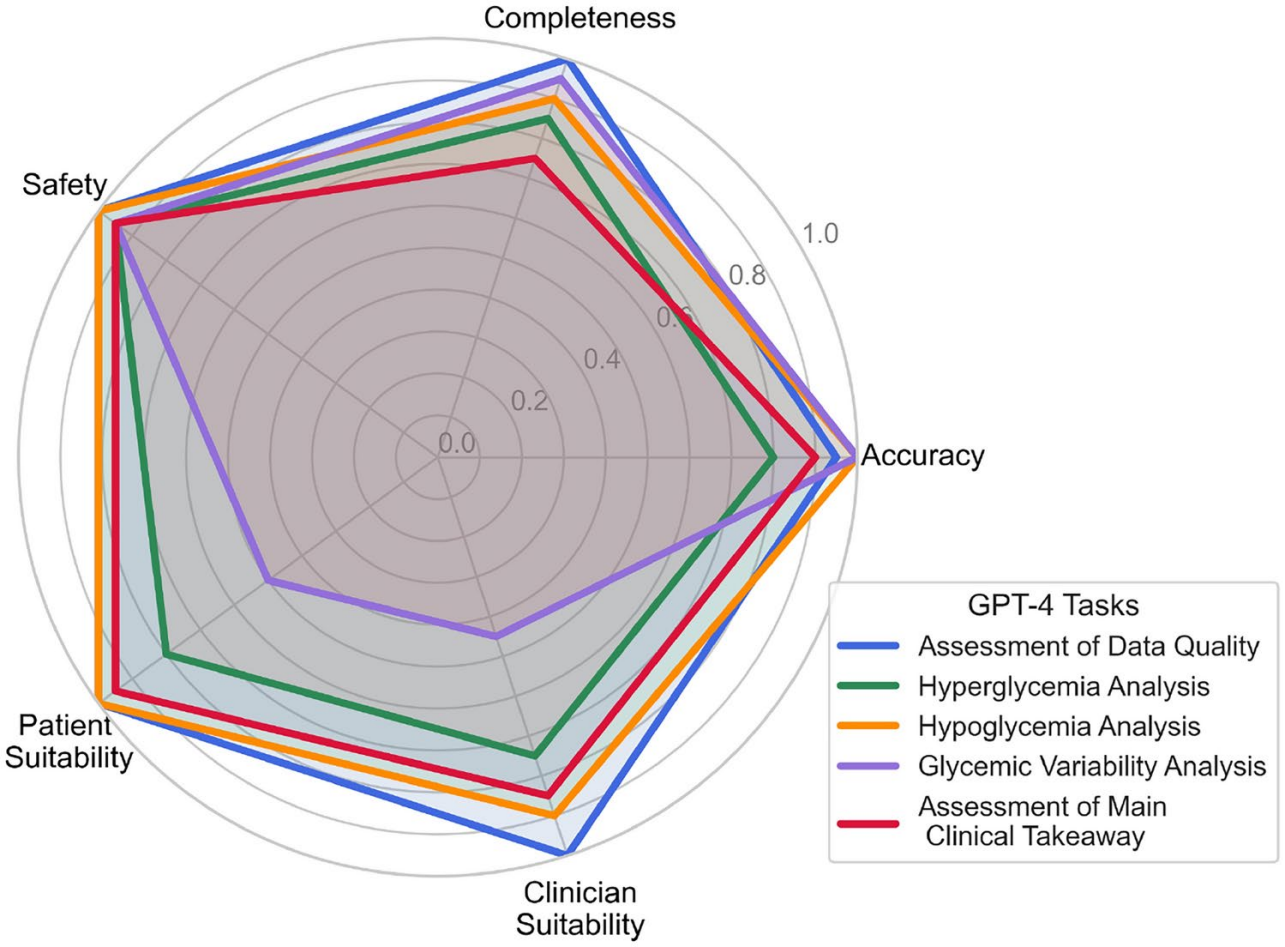
Radar plot of average scores across graders and cases for each task for each evaluation category.

Figure 2 shows the radar plot of the average scores for each category by task. GPT-4 scored well in accuracy across all 10 cases and tasks. Table 2 gives a breakdown of the error type and count, and an example of the error made. On inspection, in many of the cases, the cause of the error was GPT-4 misinterpreting the analysis to be performed. Other errors were caused by GPT-4 suggesting the wrong clinical conclusions, often by adding adjectives to suggest a degree of severity that was graded as incorrect. In one identified instance, GPT-4 came to the wrong conclusion by writing incorrect code. In this particular case, GPT-4 wrote code that was supposed to identify instances of prolonged hyperglycemia but instead incorrectly classified periods in the euglycemic range as prolonged hyperglycemia. Supplementary Figure S5 gives the breakdown of total scores for each case by the GMI; notably, accuracy was higher for cases with higher GMIs.

**Table 2:** Reasons and examples of GPT-4 statements found to be not accurate (left) and not safe (right).

Accuracy Error Examples		Safety Error Examples	
**Error type and count**	**Example**	**Error type and count**	**Example**
Error in code (1)	Output stated severe hyperglycemia occurred at a time when it did not, due to an error in the code	Implies glucose control is stable (1)	When providing a summary of glycemic variability, output described glucose control as “relatively stable” but missed that this was persistently in the hyperglycemic range
Misinterpretation of code to compute (3)	GPT-4 computed average blood glucose across a time window to identify a trend instead of looking at the time window where the average BG was maximal	Overstates hyperglycemia (2)	For a patient with good control, output stated that hyperglycemia was a concern, which could cause a patient to overcorrect into hypoglycemia
Suggests wrong clinical conclusion (3)	Output described hyperglycemia as a “notable concern” despite the fact that BG levels were only greater than 180 mg/dL on three days for a brief period		

The average scores across tasks for completeness were similar to accuracy, ranging from an average of 7.5/10 for the main takeaway to 10/10 for the data quality. There was notably good concordance between clinicians, with Gwet’s AC1 scores of 0.84, 0.83, and 0.94 for accuracy, completeness, and safety, respectively. Supplementary Table S4 quantifies the instances where both clinicians, one clinician, or no clinician supported the model summary. The primary causes of low scoring for this section were not picking up a specific trend or an instance that the clinician would have identified. For the main clinical takeaway task, notable errors were in not mentioning instances of hypoglycemia and not emphasizing nocturnal events. The scores for the safety evaluation were generally positive, with three instances of safety concerns being identified across two main error types, as listed in Table 2. The full breakdown of error types and rater agreement for accuracy, completeness, and safety is given in Supplementary Figure S6.

The scores for patient suitability and clinician suitability were variable among raters. The Gwet’s AC1 scores for these categories were 0.65 and 0.43, respectively, suggesting higher discordance between how each clinician regarded whether the GPT-4 output was useful information to communicate to the patient or for their own analysis. This was partly because the output of GPT-4 for each task was extensive, which introduced more subjectivity into grading, especially when some sentences were helpful and others were not. There were three primary reasons for low scoring and discordance for both suitability questions. GPT-4 would often highlight an event that was inconsistent with what the clinician would highlight (comprising 13% and 33% of low scores for patient suitability and clinician suitability, respectively). Similarly, low scores were caused by disagreement with the clinical conclusion that GPT-4 was suggesting (comprising 20% and 19% of low scores for patient suitability and clinician suitability, respectively). Lastly, the clinicians had different preferences on the utility of the glycemic variability analysis, and so there was high discordance for both the patient and clinician suitability evaluation for that task (comprising 67% and 48% of low scores for patient suitability and clinician suitability, respectively). The full output from GPT-4 for each case is in the Supplementary Material.

## Discussion

This work evaluated the potential of LLMs to be integrated into diabetes care through automated CGM data summaries. In this case study, we have highlighted both the potential benefits of this technology and the current limitations, and we suggest priorities for future research in this area.

Unsurprisingly, GPT-4 performed well with the metric computation tasks in part 1. When given a clear data analysis task, GPT-4 was able to load the CGM data and compute the appropriate metric in most cases. In part 2, for the open-ended data analysis prompts, for AGP summarization tasks, GPT-4 occasionally wrote code that misinterpreted the defined task. In one identified instance in the study, GPT-4 wrote code that, when executed, did not compute what was intended and, therefore, produced an inaccurate result. It should be noted that while this was the only identified error in the code construction, it is possible that other errors were made that were not identified. However, this is an issue that could potentially be addressed with enhanced prompt design, or through clearer instructions on how to write code for a specific task. The exact code written by the model for the same task varied with each case. Notably, despite this variation, the model was able to arrive at the correct answers for most of the metric generation tasks. Future work should explore how code variability changes at different temperatures. Lastly, there were periods of missing data in some of the cases that occurred during periods of prolonged hyperglycemia. Notably, GPT-4 did not impute missing data and did not infer that the missing block of data was likely all in the hyperglycemic range, as a clinician looking at the data would. It is possible that this problem could be overcome by including instructions on how to handle missing data in the prompt.

GPT-4 was able to perform useful data analysis on CGM data and integrate information about guidelines for AGP interpretation into the responses. The prompts were not designed to provide personalized recommendations to patients, so there was limited information included in the prompts about targets for glycemic control. Because of this, we found GPT-4 had several shortcomings when translating findings into clinical significance. Most notably, it did not incorporate metrics such as GMI and time in range (TIR) into the final main takeaway, and in some cases suggested that a patient with overall excellent glycemic control should treat hyperglycemia more aggressively, despite having very few episodes of hyperglycemia. Additionally, when analyzing hyperglycemia, GPT-4 did not incorporate the value that clinical concern increases for glucose measurements above the 250 mg/dL threshold compared to those above the 180 mg/dl threshold. Similarly, GPT-4 frequently highlighted instances of mild hyperglycemia that lacked clinical relevance and, at times, missed instances of brief nocturnal hypoglycemia, including instances where overnight blood glucose approached the hypoglycemic threshold. Nocturnal hypoglycemia is a significant health concern, and clinicians prioritize reviewing past instances with their patients when reviewing retrospective CGM data. Future work should investigate refined prompt design or reinforcement learning from human feedback to incorporate these values into the model. In many instances, there was discordance between the clinicians about the interpretation. This was seen across all metrics but more often in the patient suitability and clinician suitability tasks. The output of GPT-4 for each task was often long and in paragraph form. This potentially introduced a degree of subjectivity, as there were many components of the interpretation that may have stood out to different readers. Lastly, the adult and pediatric clinicians care for different patient populations, which potentially influenced the ratings of suitability, as their own interpretation styles may vary.

There were technical limitations of this study that we want to highlight. The data used for each case was simulated using different meal and bolus patterns. However, many scenarios were not simulated in the data. Notably, there were no scenarios in which patients experienced hypoglycemia and consumed rescue carbohydrates as treatment. Additionally, technical artifacts such as compression-induced hypoglycemia, extended periods of missing data, and varying calibration accuracy throughout a sensor’s lifecycle were not modeled.

This work demonstrates the potential for using LLMs in diabetes care to streamline CGM analysis. This technology could be particularly helpful in busy endocrine practices as a first pass at distilling large amounts of CGM data. The clinician could then confirm the output and build upon it to guide discussions with patients and subsequent recommendations. This technology also has the potential to be useful to primary care providers, who may have less formal training and experience with CGM and AGPs but still may be tasked with interpretation and summarization of the data for their patients. Although LLMs are not yet at the level of performance to be able to substitute for clinician input, this work demonstrates the potential of using LLMs to interpret large amounts of medical time series data.

## Supplementary Information

Supplementary Information.

## Data Availability

This work was done using synthetic data and accordingly, all CGM data is publicly available. The CSV files are available on https://github.com/lizhealey/GPT4_CGM_Summarization.git.
