# Supplementary material for: A case study on using a large language model to analyze continuous glucose monitoring data
Source: Sci Rep. 2025 Jan 7;15:1143. doi: 10.1038/s41598-024-84003-0 (PMC11707017; doi:10.1038/s41598-024-84003-0)
Supplement: Supplementary file 1 — Supplementary Information. [file 41598_2024_84003_MOESM1_ESM.pdf]

# Supplementary Material

*Supplementary Material* ........ 1

I. Data Generation ........ 1

II. Full Prompts ........ 2

III. Ambulatory Glucose Profiles ........ 4

IV. Metric Computation Task Results ........ 6

V. AGP Summarization Task Results ........ 6

VI. GPT-4 Output ........ 8

VII. Temperature Variation Experiments ........ 42

## I. Data Generation

The generated data was sampled every 5 minutes. We created 10 different 14-day scenarios using differing random carbohydrate intake and basal/bolus insulin regimens. The characteristics of the 10 cases are given in Table 1. The corresponding GMI values range from 6% to 9%. CGM data missingness was generated randomly to make the data more realistic.

We applied a minimal amount of pre-processing of the data to simplify the analysis steps that GPT-4 had to do. We converted the data for each case into a comma-separated values (CSV) file with columns for CGM value, time of day, the day of the week, and the datetime structure with the time and day together. All CGM values were rounded to the nearest 5-minute interval.

| | Mean and standard deviation across cohort | Minimum of cohort | Maximum of cohort |
|---|---|---|---|
| Mean (mg/dL) | 164.4 (40.85) | 112.6 | 237.8 |
| GMI (%) | 7.2 (1.0) | 6.0 | 9.0 |
| CV (%) | 0.3 (0.1) | 0.2 | 0.4 |
| Minimum (mg/dL) | 55.3 (9.0) | 39.0 | 70.0 |
| Maximum (mg/dL) | 337.1 (85.3) | 208.0 | 400.0 |
| Percent time active (%) | 92.7 (4.5) | 86.1 | 99.2 |
| Time in range (%) | 67.0 (27.2) | 28.0 | 99.2 |
| Percent time above 180 mg/dL (%) | 31.6 (0.274) | 0.6 | 71.2 |
| Percent time above 250 mg/dL (%) | 10.6 (0.13) | 0.0 | 41.9 |
| Percent time below 70 mg/dL (%) | 0.8 (1.0) | 0.0 | 3.1 |
| Percent time below 54 mg/dL (%) | 0.1 (0.2) | 0.0 | 0.8 |

| |
|---|
| **Table S1: Characteristics of 10 Cases**<br>GMI- Glucose Management Indicator; CV- coefficient of variation |

## II. Full Prompts

In the first part, the metric generation prompts were given as a single prompt. In the second part, the summarization tasks were prompted individually on a single thread.

### Prompt for Metric Computation Tasks:

*** Upload CSV ***

You are an endocrinologist analyzing CGM data for a patient with type 1 diabetes and on insulin therapy.
Note that the target glucose is between 70 mg/dL and 180 mg/dL, and severe hypoglycemia is defined as less than 54 mg/dL and severe hyperglycemia is defined as greater than 250 mg/dL.
Use the CGM data to answer the following questions:

Report the following metrics:
1. Number of days the sensor is active
2. % of sensor data captured
3. Mean glucose concentration
4. Glucose management indicator
5. Glucose variability (coefficient of variation)
6. % Time above target glucose range (TAR) in level 2 (>250)
7. % Time above target glucose range (TAR) in level 1( >180)
8. % Time in range (70-180 mg/dL)
9. % Time below target glucose range (TBR) in level 1 (<70)
10. % Time below target glucose range (TBR) in level 2 (<54)

Produce a list with these 10 values in it.

*** Record Output***

### Prompts for AGP Summarization Tasks:

*** Upload CSV ***

You are an endocrinologist analyzing CGM data for a patient with type 1 diabetes and on insulin therapy.
Note that the target glucose is between 70mg/dL and 180 mg/dL, and severe hypoglycemia is defined as less than 54mg/dL and severe hyperglycemia is defined as greater than 250 mg/dL.
Use the CGM data to answer the following questions:

1. (Quality of data)Assess the quality of this data noting the following factors. Is this data good quality?
- The recommended period of analysis is 14 days, if the data has less than 14 unique days of data, recommend looking at the data for more time.
- The recommended percentage of CGM activity time is greater than 70%. If there is less than 70% active time, note that the quality of this report might be impaired.

Produce an Output Summary: In a sentence, describe if this data is good quality and why

*** Record Output***

Data Analysis Instructions:
The following steps may be useful for hyperglycemia analysis:
- Note that CGM is sampled every 5 minutes
- Break the day up into 4-hour time windows to help the patient identify daily patterns, and in the final answer convert the time windows to general times of the day: “Late night”, “early morning”, “morning”, “afternoon”, “evening”, “night”.

a. (hyperglycemia) Analyze the data for hyperglycemia:

Consider whether hyperglycemia appears to be an issue with this patient. Severe hyperglycemia warrants particular concern. While there is no set target for time spent above 180%, guidance says target range should be above 70% and ideally greater. Identify whether or not there are notable:

1. Interday trends: identity if certain days stood out as having very high levels of glucose, or a very high percentage of the day spent above either hyperglycemic threshold. Mentioning the date will help the patient identify if they did something then that led to the hyperglycemia.
2. Intraday trends: take the average glucose across the 24-hour day and identify the maximum glucose in that time window and if its hyperglycemia, make note of the trend
3. Prolonged hyperglycemia: Are there prolonged high-glucose events with periods of severe hyperglycemia where glucose readings are over 250 for an hour or more (12 consecutive readings)? If so, give the days and times of day that these occurred. This will be helpful for the patient in recalling behavioral patterns.

No need to print out tables

*** Submit and Pause for Analysis ***

Produce an Output Summary: Produce a 2-6 sentence summary of the hyperglycemia analysis with key takeaways with interday trends, intraday trends, and prolonged hyperglycemia. If hyperglycemia does not seem to be a significant problem, the summary can be short. Highlight examples from the data that may be useful to say to the patient and draw attention to the dates and times of the prolonged hyperglycemia events if they exist.

*** Submit and Record Output***

b. (Hypoglycemia) Analyze the data for hypoglycemia:
According to guidelines, time spent below 70 should be under 1 hour a day, and time spent below 54 should be under 15 minutes a day.
1. Interday trends: identity if certain days stood out as having very low levels of glucose (below 70mg/dL), or a very high percentage of the day spent below either hypoglycemic threshold. Mentioning the date will help the patient identify if they did something then that led to the hypoglycemia.
2. Intraday trends: Does hypoglycemia tend to occur at certain times of the day for this patient?
3. Prolonged hypoglycemia: Are there prolonged periods of hypoglycemia where glucose stays below 70 for more than 60 minutes, or below 54 for more than 15 minutes? What day and time did these occur?

*** Submit and Pause for Analysis ***
Produce an Output Summary: Produce a 2-6 sentence summary of the hypoglycemia analysis with key takeaways with interday trends, intraday trends, and prolonged hypoglycemia. If hypoglycemia does not seem to be a significant problem, the summary can be short. Highlight examples from the data that may be useful to say to the patient and draw attention to the dates and times of the prolonged events if they exist.

*** Submit and Record Output***

c. (Glycemic variability) Analyze glycemic variability in the dataset by looking at the coefficient of variation and the daily interquartile range.
According to guidelines, the goal is to have the coefficient of variation less than 36%
1. Did any days stand out with a high coefficient of variation?
2. Look at the interquartile range over a 24-hour period. Are there certain times of the day where the interquartile range is larger?

*** Submit and Pause for Analysis ***

Produce an Output Summary: Produce a 2-6 sentence summary of the glycemic variability analysis with key takeaways with days with high variation and times of day where IQR is highest.

***Submit and Record Output***

Based on the analysis and guidelines, in a few sentences what is the primary clinical concern, if any?

*** Submit and Record Output***

## III. Ambulatory Glucose Profiles

### Case 1:

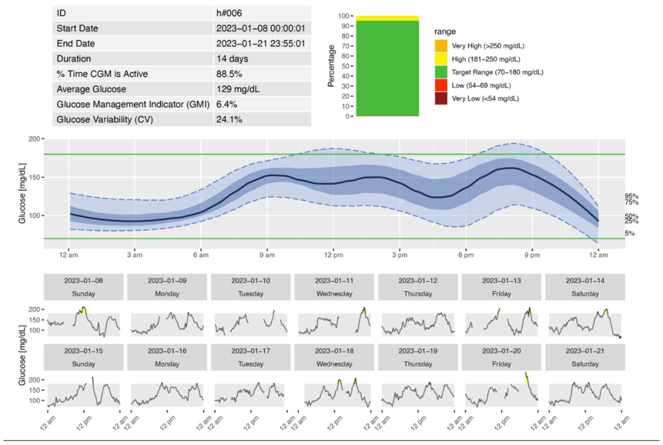


### Case 6:

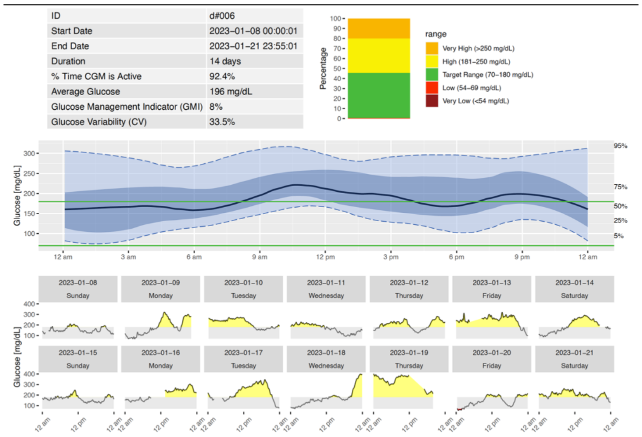


### Case 2:

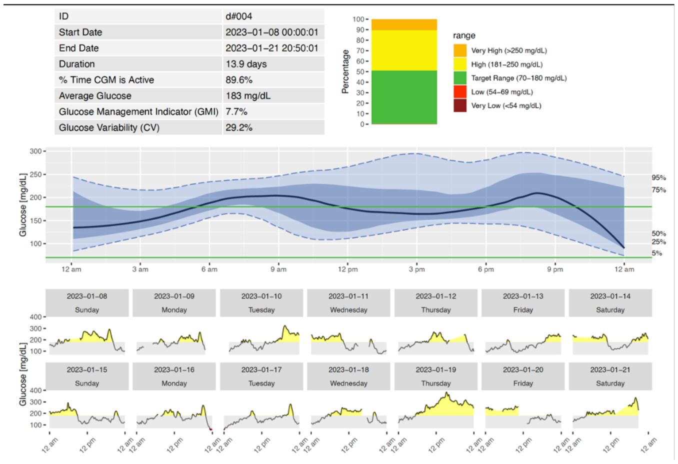


### Case 7:

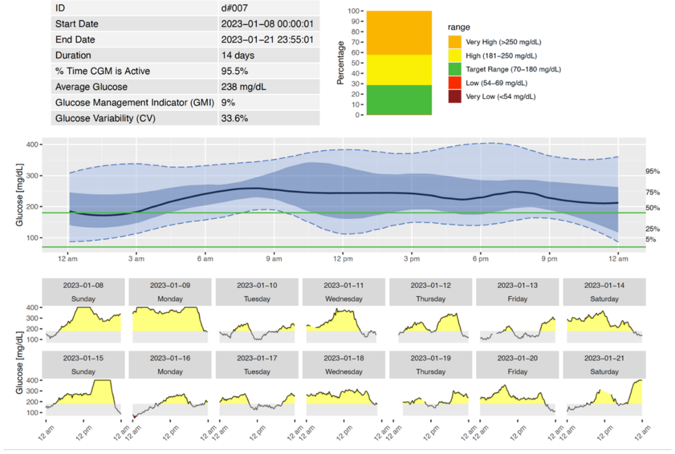


### Case 3:

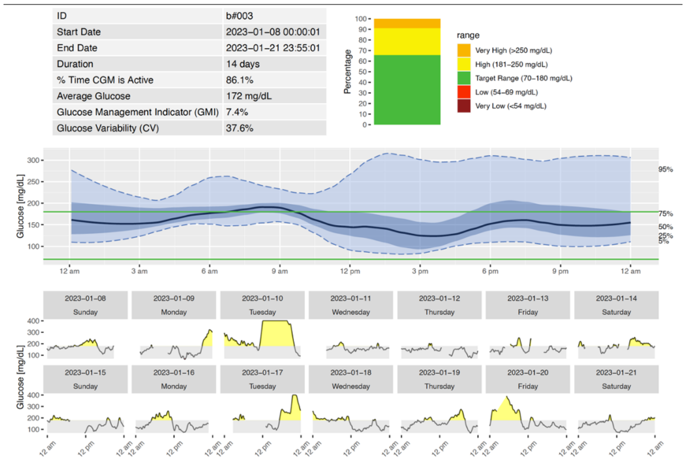


### Case 8:

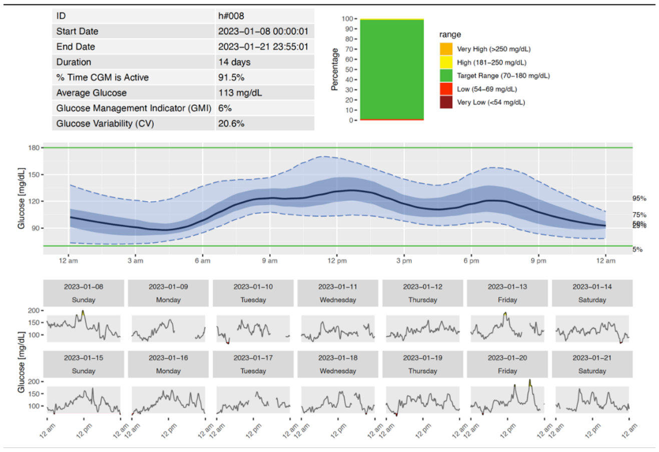


### Case 4:

### Case 9:

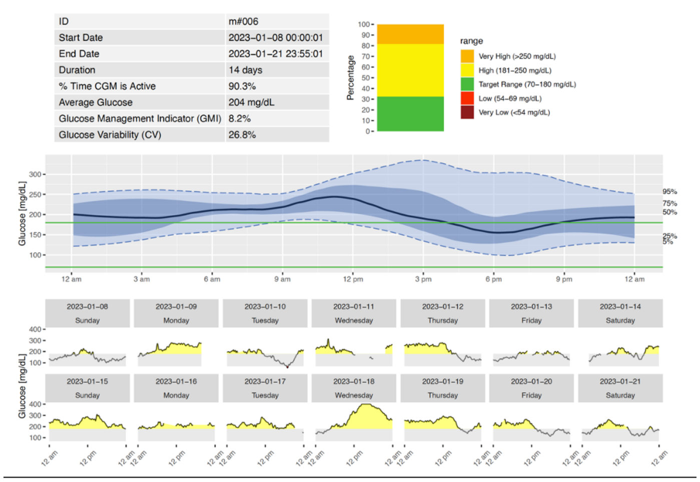


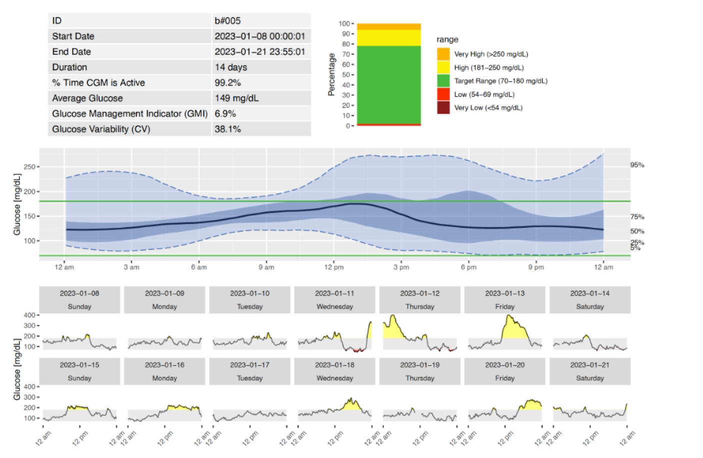


## Case 5:

## Case 10:

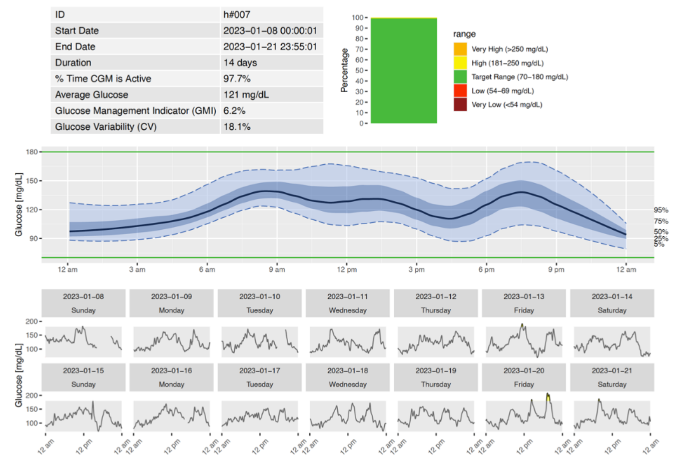


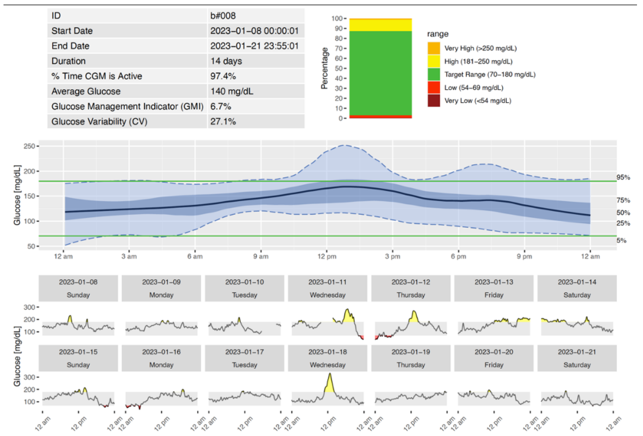


**Figure S2: Ambulatory Glucose Profiles of the 10 Cases**

## IV. Metric Computation Task Results

Table S3 shows the accuracy of the model for each of the 10 different tasks across the 10 different cases. The model performs nine out of the ten tasks with perfect accuracy. For four different cases, the GPT-4 incorrectly computes TAR. On inspection, the cause of this error in all four cases was GPT-4 computing the percentage time spent above 180 mg/dL, excluding the time spent in TAR2. See section IV for the code that was generated on each iteration.

| **Metric** | **Accuracy** | **Reason for error** |
|---|---|---|
| Number of days the sensor was active | 100% | |
| % of sensor data captured | 100% | |
| Mean glucose value | 100% | |
| Glucose Management Indicator | 100% | |
| Coefficient of variation | 100% | |
| Time above 250 mg/dL | 100% | |
| Time above 180 mg/dL | 60% | GPT-4 computed percentage spent above 180 mg/dL and below or equal to 250 mg/dL, instead of showing the percentage spent above 180 mg/dL. |
| Time between 70 mg/dL and 180 mg/dL | 100% | |
| Time below 70 mg/dL | 100% | |
| Time below 54 mg/dL | 100% | |

**Table S3: Quantitative Results Performance**

## V. AGP Summarization Task Results

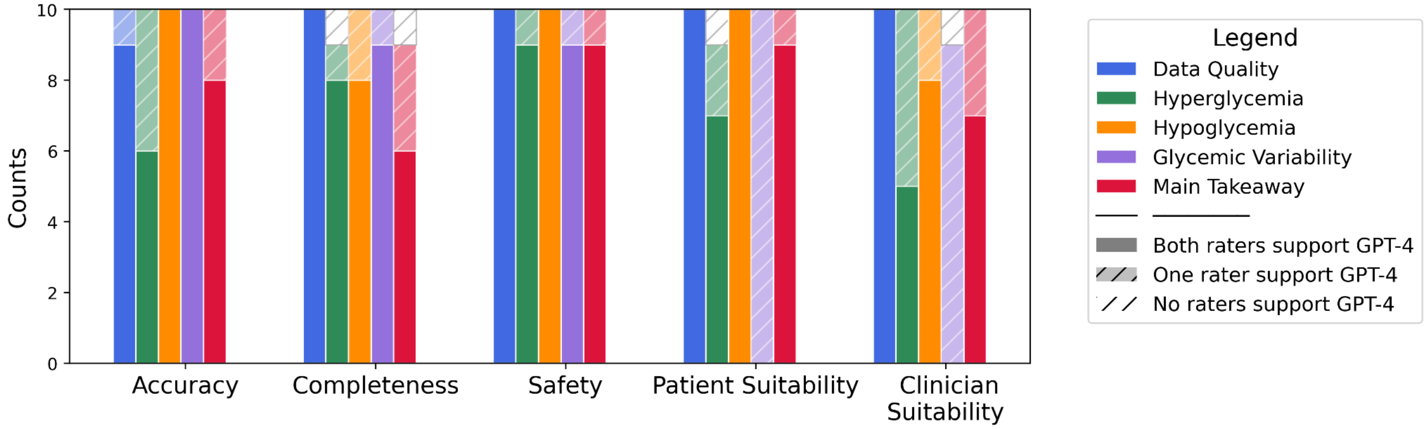


**Figure S4: Aggregate scores by category and task**

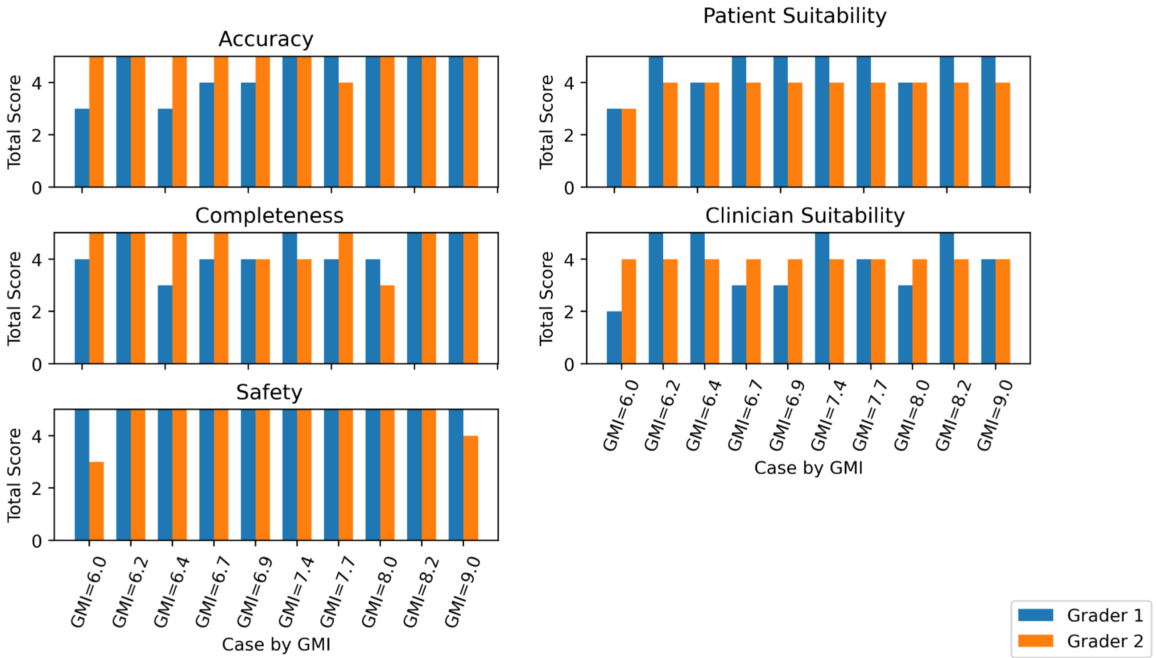


**Figure S5: Scores per case and grader by Glucose Management Indicator (GMI) across all 5 categories**

| | **Accuracy**<br>Average Score and Error Type Breakdown | **Completeness**<br>Average Score and Error Type Breakdown | **Safety**<br>Average Score and Error Type Breakdown |
|---|---|---|---|
| **Data Quality:** | 9.5 - Misinterpretation of code to compute | 10 | 10 |
| **Hyperglycemia** | 8 - Misinterpretation of code to compute<br>- Suggest wrong clinical conclusion (2)<br>- Error in code | 8.5 - Does not identify hyperglycemia trend<br>- No comment on episode of severe hyperglycemia (2) | 9.5 - Overstates hyperglycemia |
| **Hypoglycemia** | 10 | 9 - Does not specify date episode of prolonged hypoglycemia started<br>- Does not mention instances of hypoglycemia | 10 |
| **Glycemic Variability** | 10 | 9.5 - Does not specify date of high CV | 9.5 - Implies glucose control is stable |
| **Main takeaway** | 9 - Misinterpretation of code to compute<br>- Suggests wrong clinical conclusion | 7.5 - Fails to mention pattern of hypoglycemia<br>- No comment on episode of prolonged hypoglycemia (2)<br>- Fails to highlight nocturnal hyperglycemia<br>- Fails to comment on nocturnal hypoglycemia | 9.5 - Overstates hyperglycemia |
| Rater Agreeability | 0.84 | 0.83 | .94 |

**Table S6: Error breakdown for Accuracy, Completeness, and Safety with rater agreeability given by Gwet's AC1 score.**

# VI. GPT-4 Output

## Metric Generation Task Prompt Output

Responses generated on February 15th, 2024 using OpenAI's Chat GPT Data Analyst Plugin with default parameters https://chat.openai.com/g/g-HMNcP6w7d-data-analyst .

| Case | GPT-4 Output |
|---|---|
| Case 1 | To analyze the Continuous Glucose Monitoring (CGM) data for the patient with type 1 diabetes, I'll start by examining the content of the uploaded file to understand its format and the data it contains. This will allow me to calculate the requested metrics accurately. Let's proceed with loading and reviewing the data.<br><br>The CGM data includes columns for glucose readings (CGM), time of the reading (time and Time), and the day of the week (weekday). To calculate the requested metrics, I'll use the CGM and Time columns, where Time appears to combine the date and time of each reading.<br><br>Let's proceed with the calculations for:<br><br>Number of days the sensor is active<br>% of sensor data captured<br>Mean glucose concentration<br>Glucose management indicator (GMI)<br>Glucose variability (coefficient of variation, CV)<br>% Time above target glucose range (TAR) in level 2 (>250 mg/dL)<br>% Time above target glucose range (TAR) in level 1 (>180 mg/dL)<br>% Time in range (70-180 mg/dL)<br>% Time below target glucose range (TBR) in level 1 (<70 mg/dL)<br>% Time below target glucose range (TBR) in level 2 (<54 mg/dL)<br>I'll start by preparing and analyzing the data to compute these metrics.<br><br>The analysis of the CGM data for the patient with type 1 diabetes has yielded the following metrics:<br><br>Number of days the sensor is active: 14 days<br>% of sensor data captured: 88.54%<br>Mean glucose concentration: 128.56 mg/dL<br>Glucose management indicator: 6.39<br>Glucose variability (coefficient of variation): 24.11%<br>% Time above target glucose range (TAR) in level 2 (>250 mg/dL): 0.0%<br>% Time above target glucose range (TAR) in level 1 (>180 mg/dL, exclusive of >250): 4.71%<br>% Time in range (70-180 mg/dL): 95.01%<br>% Time below target glucose range (TBR) in level 1 (<70 mg/dL): 0.28%<br>% Time below target glucose range (TBR) in level 2 (<54 mg/dL): 0.0%<br>These metrics provide a comprehensive view of the glucose management for this patient, indicating good time in range and low occurrences of hypoglycemia and hyperglycemia. |
| Case 2 | To provide you with the requested metrics from the CGM (Continuous Glucose Monitoring) data, I'll first need to examine the contents of the file you uploaded. I will start by loading and reviewing the data to understand its structure. This will allow me to accurately calculate the metrics you've requested. Let's begin by loading the file and taking a quick look at its contents.<br><br>The CGM data includes several columns: an unnamed column which appears to be an index, glucose concentration (CGM), time of the day, weekday, and a timestamp combining date and time. To calculate the requested metrics, I'll primarily focus on the CGM values and the timestamp to determine the duration of sensor activity, calculate the mean glucose concentration, and assess glucose management indicators and time in various glucose ranges.<br><br>Let's proceed with the calculations for each of the requested metrics.<br><br>Here are the calculated metrics based on the CGM data provided:<br><br>Number of days the sensor is active: 14 days<br>% of sensor data captured: 88.81%<br>Mean glucose concentration: 183.26 mg/dL<br>Glucose management indicator (GMI): 7.69<br>Glucose variability (coefficient of variation): 29.22%<br>% Time above target glucose range (TAR) in level 2 (>250 mg/dL): 10.47%<br>% Time above target glucose range (TAR) in level 1 (>180 mg/dL): 48.81% (This includes time above 250 mg/dL)<br>% Time in range (70-180 mg/dL): 50.80%<br>% Time below target glucose range (TBR) in level 1 (<70 mg/dL): 0.39%<br>% Time below target glucose range (TBR) in level 2 (<54 mg/dL): 0.00%<br>These metrics provide a comprehensive overview of the glucose management status for the patient, indicating areas where management strategies could potentially be optimized. |
| Case 3 | To provide you with the requested metrics, I will first analyze the uploaded CGM (Continuous Glucose Monitoring) data file. I'll start by loading the data to understand its structure, which will allow us to calculate the metrics accurately. Let's proceed with loading and reviewing the data.<br><br>The CGM data consists of columns indicating glucose measurements (CGM), the time of measurement, the day of the week, and a combined date-time format. With this structure in mind, I'll proceed to calculate the requested metrics. Let's start by computing each of the metrics step by step. |

| | |
|---|---|
| | Based on the analysis of the CGM data, here are the calculated metrics:<br><br>Number of days the sensor is active: 14 days<br>% of sensor data captured: 86.06%<br>Mean glucose concentration: 171.67 mg/dL<br>Glucose Management Indicator (GMI): 7.42%<br>Glucose variability (coefficient of variation): 37.59%<br>% Time above target glucose range (TAR) in level 2 (>250 mg/dL): 8.62%<br>% Time above target glucose range (TAR) in level 1 (>180 mg/dL): 25.50%<br>% Time in range (70-180 mg/dL): 65.76%<br>% Time below target glucose range (TBR) in level 1 (<70 mg/dL): 0.12%<br>% Time below target glucose range (TBR) in level 2 (<54 mg/dL): 0.00%<br>These metrics provide a comprehensive view of the patient's glucose levels over the period the sensor was active, including times of hyperglycemia, hypoglycemia, and periods within the target range. |
| Case 4 | To proceed with the analysis of the CGM (Continuous Glucose Monitoring) data and answer your questions, I'll first need to load and examine the content of the uploaded file. This will involve checking the structure of the data, including the date range, frequency of glucose measurements, and the glucose values themselves. Let's start by loading the data from the uploaded file and take a quick look at its structure.<br><br>The CGM data consists of several columns, including an unnamed index column, CGM glucose values, time of the measurement, the day of the week, and a combined date and time column labeled "Time". To answer your questions, we'll focus primarily on the "CGM" values and the "Time" column to calculate the metrics you've requested.<br><br>Let's proceed with the analysis to report the requested metrics:<br><br>Number of days the sensor is active<br>% of sensor data captured<br>Mean glucose concentration<br>Glucose management indicator (GMI)<br>Glucose variability (coefficient of variation)<br>% Time above target glucose range (TAR) in level 2 (>250)<br>% Time above target glucose range (TAR) in level 1 (>180)<br>% Time in range (70-180mg/dL)<br>% Time below target glucose range (TBR) in level 1 (<70)<br>% Time below target glucose range (TBR) in level 2 (<54)<br>First, we need to preprocess the data, especially the "Time" column, to extract the dates and calculate the number of active sensor days and other time-based metrics. Let's start with these calculations.<br><br>Based on the analysis of the CGM data, here are the metrics you requested:<br><br>Number of days the sensor is active: 14 days<br>% of sensor data captured: 90.33%<br>Mean glucose concentration: 204.30 mg/dL<br>Glucose management indicator (GMI): 8.20<br>Glucose variability (coefficient of variation): 26.82%<br>% Time above target glucose range (TAR) in level 2 (>250 mg/dL): 18.29%<br>% Time above target glucose range (TAR) in level 1 (>180 mg/dL, excluding >250): 49.15%<br>% Time in range (70-180 mg/dL): 32.43%<br>% Time below target glucose range (TBR) in level 1 (<70 mg/dL): 0.14%<br>% Time below target glucose range (TBR) in level 2 (<54 mg/dL): 0.00%<br>These metrics provide a comprehensive overview of the glucose management for this patient, indicating areas where adjustments might be necessary to improve time in range and reduce high glucose levels. |
| Case 5 | The data consists of columns for an unnamed identifier, Continuous Glucose Monitoring (CGM) values, time, weekday, and a datetime stamp. I'll use this information to calculate the requested metrics.<br><br>Number of days the sensor is active: This can be determined by the range of dates in the "Time" column.<br>% of sensor data captured: Assuming the sensor should capture data every 5 minutes, I can calculate this based on the total entries vs. the expected number of entries.<br>Mean glucose concentration: The average of the "CGM" values.<br>Glucose management indicator (GMI): Calculated using the formula<br>GMI=3.31+0.02392×mean glucose mg/dL.<br>Glucose variability (coefficient of variation): Standard deviation of "CGM" values divided by the mean, multiplied by 100.<br>% Time above target glucose range (TAR) in level 2 (>250).<br>% Time above target glucose range (TAR) in level 1 (>180).<br>% Time in range (70-180 mg/dL).<br>% Time below target glucose range (TBR) in level 1 (<70).<br>% Time below target glucose range (TBR) in level 2 (<54).<br>Let's proceed to calculate these metrics.<br><br>The analysis of the CGM data yields the following metrics:<br><br>Number of days the sensor is active: 14 days<br>% of sensor data captured: 97.67%<br>Mean glucose concentration: 121.24 mg/dL<br>Glucose management indicator (GMI): 6.21<br>Glucose variability (coefficient of variation): 18.12%<br>% Time above target glucose range (TAR) in level 2 (>250): 0.0%<br>% Time above target glucose range (TAR) in level 1 (>180) (exclusive): 0.63%<br>% Time in range (70-180mg/dL): 99.37%<br>% Time below target glucose range (TBR) in level 1 (<70): 0.0%<br>% Time below target glucose range (TBR) in level 2 (<54): 0.0% |

| | |
|---|---|
| | These values provide a comprehensive overview of the glucose management for this patient over the analyzed period. |
| Case 6 | To accurately report the requested metrics from the provided Continuous Glucose Monitoring (CGM) data, I'll start by examining the structure of the uploaded CSV file to understand its format, including how the glucose readings and timestamps are recorded. This will enable me to calculate:<br><br>The number of days the sensor was active.<br>The percentage of sensor data captured (assuming we know the expected data capture rate).<br>The mean glucose concentration.<br>The Glucose Management Indicator (GMI), which can be estimated from the mean glucose concentration.<br>The glucose variability, as measured by the coefficient of variation.<br>The percentage of time above the target glucose range (TAR) in level 2 (>250 mg/dL).<br>The percentage of time above TAR in level 1 (>180 mg/dL).<br>The percentage of time in range (TIR) (70-180 mg/dL).<br>The percentage of time below the target glucose range (TBR) in level 1 (<70 mg/dL).<br>The percentage of time below TBR in level 2 (<54 mg/dL).<br>Let's start by loading and examining the content of the file to understand its structure.<br><br>The CGM data file contains several columns, including:<br><br>Unnamed: 0: Appears to be an index column.<br>CGM: Glucose concentration in mg/dL.<br>time: Time of day for the reading.<br>weekday: Day of the week for the reading.<br>Time: Date and time of the reading, combining date with the time column.<br>To calculate the requested metrics, I'll primarily use the CGM and Time columns. The Time column will help in determining the number of days the sensor was active and other time-related metrics. Let's proceed with calculating the requested metrics.<br><br>Based on the analysis of the Continuous Glucose Monitoring (CGM) data, here are the requested metrics:<br><br>Number of days the sensor was active: 14 days<br>Percentage of sensor data captured: 92.41%<br>Mean glucose concentration: 195.55 mg/dL<br>Glucose Management Indicator (GMI): 7.99<br>Glucose variability (coefficient of variation): 33.46%<br>Percentage of Time above target glucose range (TAR) in level 2 (>250 mg/dL): 19.78%<br>Percentage of Time above TAR in level 1 (>180 mg/dL, exclusive of >250 mg/dL): 34.49%<br>Percentage of Time in range (70-180 mg/dL): 45.20%<br>Percentage of Time below target glucose range (TBR) in level 1 (<70 mg/dL): 0.54%<br>Percentage of Time below TBR in level 2 (<54 mg/dL): 0.00%<br>These metrics provide a comprehensive overview of the patient's glucose management over the 14-day period. |
| Case 7 | To provide the requested metrics from the Continuous Glucose Monitoring (CGM) data, I will start by loading and examining the contents of the uploaded CSV file. This will allow me to understand the data structure and proceed with the analysis accordingly. Let's begin by loading the data.<br><br>The CGM data contains columns for an index ("Unnamed: 0"), glucose concentration ("CGM"), time of day ("time" and "Time"), and the day of the week ("weekday"). It appears that "Time" includes both the date and time, making it suitable for our analysis.<br><br>Next, I will calculate the requested metrics based on this data. These calculations will include:<br><br>Number of days the sensor is active.<br>Percentage of sensor data captured.<br>Mean glucose concentration.<br>Glucose Management Indicator (GMI).<br>Glucose variability (coefficient of variation).<br>Percentage of time above target glucose range in level 2 (>250 mg/dL).<br>Percentage of time above target glucose range in level 1 (>180 mg/dL).<br>Percentage of time in range (70-180 mg/dL).<br>Percentage of time below target glucose range in level 1 (<70 mg/dL).<br>Percentage of time below target glucose range in level 2 (<54 mg/dL).<br>Let's proceed with these calculations.<br><br>The calculated metrics from the CGM data are as follows:<br><br>Number of days the sensor is active: 14 days<br>Percentage of sensor data captured: 95.49%<br>Mean glucose concentration: 237.82 mg/dL<br>Glucose Management Indicator (GMI): 9.00<br>Glucose variability (coefficient of variation): 33.57%<br>Percentage of time above target glucose range (TAR) in level 2 (>250 mg/dL): 41.90%<br>Percentage of time above target glucose range (TAR) in level 1 (>180 mg/dL, excluding >250 mg/dL): 29.32%<br>Percentage of time in range (70-180 mg/dL): 28.44%<br>Percentage of time below target glucose range (TBR) in level 1 (<70 mg/dL): 0.34%<br>Percentage of time below target glucose range (TBR) in level 2 (<54 mg/dL): 0.08%<br>These metrics provide a comprehensive overview of the glucose control for the patient, indicating a high mean glucose concentration and significant time spent above the target glucose range. |
| Case 8 | To provide you with the requested metrics based on the CGM (Continuous Glucose Monitoring) data, I'll first need to analyze the uploaded file to understand its structure and contents. Let's start by loading and previewing the data from the CSV file you've provided. This will help us determine how to proceed with calculating the specific metrics. |

| | |
|---|---|
| | The dataset contains columns for the CGM readings, time, weekday, and a combined date-time column labeled "Time". To calculate the metrics you've requested, I'll use the "CGM" values and the "Time" column to understand the glucose concentration over time and assess the sensor's active period.<br><br>Let's proceed with calculating the requested metrics:<br><br>Number of days the sensor is active<br>% of sensor data captured<br>Mean glucose concentration<br>Glucose management indicator (GMI)<br>Glucose variability (coefficient of variation)<br>% Time above target glucose range (TAR) in level 2 (>250 mg/dL)<br>% Time above target glucose range (TAR) in level 1 (>180 mg/dL)<br>% Time in range (70-180 mg/dL)<br>% Time below target glucose range (TBR) in level 1 (<70 mg/dL)<br>% Time below target glucose range (TBR) in level 2 (<54 mg/dL)<br>I will start by preprocessing the data and then calculate each metric sequentially.<br><br>Based on the analysis of the CGM data provided, here are the calculated metrics:<br><br>Number of days the sensor is active: 14 days<br>% of sensor data captured: 91.54%<br>Mean glucose concentration: 112.59 mg/dL<br>Glucose Management Indicator (GMI): 6.00<br>Glucose variability (coefficient of variation): 20.57%<br>% Time above target glucose range (TAR) in level 2 (>250 mg/dL): 0.0%<br>% Time above target glucose range (TAR) in level 1 (>180 mg/dL): 0.92%<br>% Time in range (70-180 mg/dL): 97.78%<br>% Time below target glucose range (TBR) in level 1 (<70 mg/dL): 1.30%<br>% Time below target glucose range (TBR) in level 2 (<54 mg/dL): 0.0%<br>These metrics provide a comprehensive overview of the glucose levels and management for the patient over the 14-day period during which the sensor was active. |
| Case 9 | The CSV file contains continuous glucose monitoring (CGM) data with columns indicating the glucose measurement (CGM), time of measurement, weekday, and a combined date and time column. To analyze this data according to the specified metrics, I'll proceed with the following steps:<br><br>Convert the "Time" column to a datetime format to accurately calculate the number of days the sensor was active.<br>Calculate the total possible readings and compare it to the actual number of readings to determine the % of sensor data captured.<br>Compute the mean glucose concentration.<br>Calculate the Glucose Management Indicator (GMI) using the formula:<br><br>GMI=3.31+0.02392×mean glucose.<br>Calculate the glucose variability as the coefficient of variation, which is the standard deviation divided by the mean glucose concentration.<br>Determine the % time in various glucose ranges to answer the remaining metrics (6-10).<br>Let's start with the first step.<br><br>Based on the analysis of the continuous glucose monitoring (CGM) data, the following metrics have been calculated:<br><br>Number of days the sensor is active: 14 days<br>% of sensor data captured: 99.16%<br>Mean glucose concentration: 148.84 mg/dL<br>Glucose Management Indicator (GMI): 6.87<br>Glucose variability (coefficient of variation): 38.07%<br>% Time above target glucose range (TAR) in level 2 (>250 mg/dL): 6.05%<br>% Time above target glucose range (TAR) in level 1 (>180 mg/dL): 21.59%<br>% Time in range (70-180 mg/dL): 76.41%<br>% Time below target glucose range (TBR) in level 1 (<70 mg/dL): 2.00%<br>% Time below target glucose range (TBR) in level 2 (<54 mg/dL): 0.25%<br>These metrics provide a comprehensive overview of the patient's glucose management over the analyzed period. |
| Case 10 | To generate the requested metrics from the Continuous Glucose Monitoring (CGM) data for your analysis, I will first need to examine the data provided in the file you uploaded. I'll start by loading and inspecting the data to understand its structure and contents. Let's proceed with that step.<br><br>The dataset consists of the following columns:<br><br>An unnamed column that seems to be an index.<br>CGM: Glucose concentration in mg/dL.<br>time: Time of the day when the measurement was taken.<br>weekday: Day of the week.<br>Time: The actual date and time when the measurement was taken.<br>To proceed with the analysis, I will:<br><br>Parse the Time column to a datetime format to accurately calculate the number of days the sensor was active.<br>Calculate the metrics as requested.<br>Let's begin with these steps.<br><br>The analysis of the Continuous Glucose Monitoring (CGM) data for the patient with type 1 diabetes yields the following metrics:<br><br>Number of days the sensor is active: 14 days<br>% of sensor data captured: 97.37%<br>Mean glucose concentration: 140.25 mg/dL |

| | |
|---|---|
| | Glucose management indicator (GMI): 6.66<br>Glucose variability (coefficient of variation): 27.13%<br>% Time above target glucose range (TAR) in level 2 (>250 mg/dL): 1.22%<br>% Time above target glucose range (TAR) in level 1 (>180 mg/dL): 12.40% (includes time above 250 mg/dL)<br>% Time in range (70-180 mg/dL): 84.51%<br>% Time below target glucose range (TBR) in level 1 (<70 mg/dL): 3.08%<br>% Time below target glucose range (TBR) in level 2 (<54 mg/dL): 0.76%<br>These metrics provide valuable insights into the patient's glucose control, indicating a good percentage of time spent within the target range and relatively low percentages of severe hypoglycemia and hyperglycemia. |

**Table S7: GPT-4 Output for Quantitative Prompts**

## Metric Generation Task Code Written

| Case | Code Written |
|---|---|

Case 1

```
from datetime import datetime

# Convert 'Time' column to datetime format
cgm_data['Time'] = pd.to_datetime(cgm_data['Time'], format='%m/%d/%y %H:%M')

# Calculate the number of days the sensor is active
num_days_active = (cgm_data['Time'].max() - cgm_data['Time'].min()).days + 1

# Assuming the sensor should capture data every 5 minutes, calculate the total expected readings
expected_readings_per_day = 24 * 60 / 5
total_expected_readings = expected_readings_per_day * num_days_active

# Calculate % of sensor data captured
percent_data_captured = (len(cgm_data) / total_expected_readings) * 100

# Mean glucose concentration
mean_glucose = cgm_data['CGM'].mean()

# Glucose Management Indicator (GMI) calculation
gmi = 3.31 + (0.02392 * mean_glucose)

# Glucose variability (Coefficient of Variation, CV)
glucose_cv = (cgm_data['CGM'].std() / mean_glucose) * 100

# Calculate % Time in different glucose ranges
total_measurements = len(cgm_data)
time_above_250 = (cgm_data['CGM'] > 250).sum() / total_measurements * 100
time_above_180 = (cgm_data['CGM'] > 180).sum() / total_measurements * 100
time_in_range = ((cgm_data['CGM'] >= 70) & (cgm_data['CGM'] <= 180)).sum() / total_measurements * 100
time_below_70 = (cgm_data['CGM'] < 70).sum() / total_measurements * 100
time_below_54 = (cgm_data['CGM'] < 54).sum() / total_measurements * 100

# Adjust % Time above TAR level 1 to exclude readings above 250
time_above_180_exclusive = time_above_180 - time_above_250

metrics = [
    num_days_active,  # Number of days the sensor is active
    percent_data_captured,  # % of sensor data captured
    mean_glucose,  # Mean glucose concentration
    gmi,  # Glucose management indicator
    glucose_cv,  # Glucose variability (CV)
    time_above_250,  # % Time above TAR in level 2
    time_above_180_exclusive,  # % Time above TAR in level 1 (exclusive)
    time_in_range,  # % Time in range
    time_below_70,  # % Time below TBR in level 1
    time_below_54  # % Time below TBR in level 2
]

Metrics
```

Case 2

```
from datetime import datetime

# Convert 'Time' column to datetime format to facilitate analysis
cgm_data['Time'] = pd.to_datetime(cgm_data['Time'], format='%m/%d/%y %H:%M')

# Calculate the number of days the sensor is active
sensor_start_date = cgm_data['Time'].min().date()
sensor_end_date = cgm_data['Time'].max().date()
num_days_active = (sensor_end_date - sensor_start_date).days + 1  # Including both start and end date
```

| | |
|---|---|
| | <pre># Calculate the total possible readings (assuming 5-minute intervals throughout the day)<br>total_possible_readings = num_days_active * 24 * 60 / 5  # 288 readings per day<br><br># Calculate % of sensor data captured<br>percent_data_captured = (len(cgm_data) / total_possible_readings) * 100<br><br># Calculate mean glucose concentration<br>mean_glucose = cgm_data['CGM'].mean()<br><br># Glucose management indicator (GMI)<br>gmi = 3.31 + (0.02392 * mean_glucose)<br><br># Glucose variability (coefficient of variation)<br>coefficient_of_variation = cgm_data['CGM'].std() / mean_glucose * 100<br><br># Calculate time above, in, and below range<br>time_above_250 = len(cgm_data[cgm_data['CGM'] > 250]) / len(cgm_data) * 100<br>time_above_180 = len(cgm_data[(cgm_data['CGM'] > 180) & (cgm_data['CGM'] <= 250)]) / len(cgm_data) * 100<br>time_in_range = len(cgm_data[(cgm_data['CGM'] >= 70) & (cgm_data['CGM'] <= 180)]) / len(cgm_data) * 100<br>time_below_70 = len(cgm_data[cgm_data['CGM'] < 70]) / len(cgm_data) * 100<br>time_below_54 = len(cgm_data[cgm_data['CGM'] < 54]) / len(cgm_data) * 100<br><br># Compile metrics into a list<br>metrics = [<br>    num_days_active,  # Number of days the sensor is active<br>    percent_data_captured,  # % of sensor data captured<br>    mean_glucose,  # Mean glucose concentration<br>    gmi,  # Glucose management indicator<br>    coefficient_of_variation,  # Glucose variability (coefficient of variation)<br>    time_above_250,  # % Time above TAR in level 2 (>250)<br>    time_above_180 + time_above_250,  # % Time above TAR in level 1 (>180) including >250<br>    time_in_range,  # % Time in range (70-180mg/dL)<br>    time_below_70,  # % Time below TBR in level 1 (<70)<br>    time_below_54  # % Time below TBR in level 2 (<54)<br>]<br><br>Metrics</pre> |
| Case 3 | <pre>from datetime import datetime<br><br># Convert 'Time' column to datetime<br>cgm_data['Datetime'] = pd.to_datetime(cgm_data['Time'])<br><br># Calculate the number of days the sensor is active<br>num_days_active = (cgm_data['Datetime'].max() - cgm_data['Datetime'].min()).days + 1<br><br># Assuming the sensor captures data every 5 minutes, calculate the % of sensor data captured<br>expected_measurements_per_day = 24 * 60 / 5<br>total_expected_measurements = expected_measurements_per_day * num_days_active<br>percent_data_captured = (len(cgm_data) / total_expected_measurements) * 100<br><br># Calculate mean glucose concentration<br>mean_glucose_concentration = cgm_data['CGM'].mean()<br><br># Glucose Management Indicator (GMI) calculation, assuming the formula: GMI (%) = (3.31 + 0.02392 * Mean Glucose in mg/dL)<br>gmi = 3.31 + 0.02392 * mean_glucose_concentration<br><br># Glucose variability (coefficient of variation) is the standard deviation divided by the mean<br>glucose_variability = cgm_data['CGM'].std() / mean_glucose_concentration<br><br># Calculate % Time in different glucose ranges<br>time_above_250 = len(cgm_data[cgm_data['CGM'] > 250]) / len(cgm_data) * 100<br>time_above_180 = len(cgm_data[cgm_data['CGM'] > 180]) / len(cgm_data) * 100 - time_above_250<br>time_in_range = len(cgm_data[(cgm_data['CGM'] >= 70) & (cgm_data['CGM'] <= 180)]) / len(cgm_data) * 100<br>time_below_70 = len(cgm_data[cgm_data['CGM'] < 70]) / len(cgm_data) * 100<br>time_below_54 = len(cgm_data[cgm_data['CGM'] < 54]) / len(cgm_data) * 100<br>time_below_70 -= time_below_54<br><br>metrics = [<br>    num_days_active,<br>    percent_data_captured,<br>    mean_glucose_concentration,<br>    gmi,<br>    glucose_variability,<br>    time_above_250,<br>    time_above_180,<br>    time_in_range,<br>    time_below_70,<br>    time_below_54<br>]<br><br>Metrics</pre> |
| Case 4 | <pre>from datetime import datetime</pre> |

```
# Convert 'Time' column to datetime format
cgm_data['Datetime'] = pd.to_datetime(cgm_data['Time'], format='%m/%d/%y %H:%M')

# Determine the number of unique days the sensor was active
num_days_active = cgm_data['Datetime'].dt.date.nunique()

# Calculate the total possible data points assuming a reading every 5 minutes over the number of days
total_possible_readings = num_days_active * 24 * 60 / 5

# Calculate the actual number of readings captured
actual_readings_captured = cgm_data.shape[0]

# Calculate % of sensor data captured
percent_data_captured = (actual_readings_captured / total_possible_readings) * 100

# Calculate mean glucose concentration
mean_glucose = cgm_data['CGM'].mean()

# Glucose management indicator (GMI) calculation, formula: GMI = 3.31 + (0.02392 × Mean glucose in mg/dL)
gmi = 3.31 + (0.02392 * mean_glucose)

# Calculate glucose variability (coefficient of variation), formula: CV = (Standard deviation / Mean) * 100
cv = (cgm_data['CGM'].std() / mean_glucose) * 100

# Calculate % time in various glucose ranges
total_time_minutes = actual_readings_captured * 5  # Each reading represents 5 minutes
tar_level_2 = cgm_data[cgm_data['CGM'] > 250].shape[0] * 5 / total_time_minutes * 100
tar_level_1 = cgm_data[cgm_data['CGM'] > 180].shape[0] * 5 / total_time_minutes * 100
time_in_range = cgm_data[(cgm_data['CGM'] >= 70) & (cgm_data['CGM'] <= 180)].shape[0] * 5 / total_time_minutes * 100
tbr_level_1 = cgm_data[cgm_data['CGM'] < 70].shape[0] * 5 / total_time_minutes * 100
tbr_level_2 = cgm_data[cgm_data['CGM'] < 54].shape[0] * 5 / total_time_minutes * 100

# Adjust TAR level 1 to exclude TAR level 2 counts
tar_level_1_adjusted = tar_level_1 - tar_level_2

metrics_list = [num_days_active, percent_data_captured, mean_glucose, gmi, cv, tar_level_2, tar_level_1_adjusted,
time_in_range, tbr_level_1, tbr_level_2]

metrics_list
```

Case 5

```
from datetime import datetime

# Convert the 'Time' column to datetime format for easier manipulation
data['Time'] = pd.to_datetime(data['Time'], format='%m/%d/%y %H:%M')

# 1. Number of days the sensor is active
num_days = (data['Time'].dt.date.max() - data['Time'].dt.date.min()).days + 1

# Assuming data should be captured every 5 minutes, calculate the total expected measurements
# 288 measurements per day (24 hours * 60 minutes / 5 minutes)
expected_measurements_per_day = 288
total_expected_measurements = num_days * expected_measurements_per_day

# 2. % of sensor data captured
percent_data_captured = (len(data) / total_expected_measurements) * 100

# 3. Mean glucose concentration
mean_glucose = data['CGM'].mean()

# 4. Glucose management indicator (GMI)
GMI = 3.31 + 0.02392 * mean_glucose

# 5. Glucose variability (coefficient of variation)
glucose_cv = (data['CGM'].std() / mean_glucose) * 100

# 6-10. Time in/out of range calculations
total_time = len(data)
time_above_250 = (data['CGM'] > 250).sum() / total_time * 100
time_above_180 = (data['CGM'] > 180).sum() / total_time * 100
time_in_range = ((data['CGM'] >= 70) & (data['CGM'] <= 180)).sum() / total_time * 100
time_below_70 = (data['CGM'] < 70).sum() / total_time * 100
time_below_54 = (data['CGM'] < 54).sum() / total_time * 100

# Subtract the higher range from the total to get the exclusive percentages for TAR level 1
time_above_180_exclusive = time_above_180 - time_above_250

# List of metrics
metrics = [
    num_days,
    percent_data_captured,
    mean_glucose,
    GMI,
    glucose_cv,
    time_above_250,
    time_above_180_exclusive,
    time_in_range,
```

| | |
|---|---|
| | ```<br>    time_below_70,<br>    time_below_54<br>]<br><br>metrics<br>``` |

Case 6

```
from datetime import datetime

# Convert 'Time' column to datetime format for easier manipulation
cgm_data['Time'] = pd.to_datetime(cgm_data['Time'], format='%m/%d/%y %H:%M')

# Calculate the number of days the sensor was active
num_days = (cgm_data['Time'].max() - cgm_data['Time'].min()).days + 1

# Assuming data capture every 5 minutes, calculate % of sensor data captured
expected_readings_per_day = 24 * 60 / 5
total_expected_readings = expected_readings_per_day * num_days
percent_data_captured = (len(cgm_data) / total_expected_readings) * 100

# Calculate mean glucose concentration
mean_glucose = cgm_data['CGM'].mean()

# Calculate Glucose Management Indicator (GMI)
# GMI formula: 3.31 + (0.02392 * Mean Glucose)
gmi = 3.31 + (0.02392 * mean_glucose)

# Calculate glucose variability (Coefficient of Variation)
cv = cgm_data['CGM'].std() / mean_glucose

# Calculate % Time in various glucose ranges
total_time = len(cgm_data)
time_above_250 = (cgm_data['CGM'] > 250).sum() / total_time * 100
time_above_180 = (cgm_data['CGM'] > 180).sum() / total_time * 100
time_in_range = ((cgm_data['CGM'] >= 70) & (cgm_data['CGM'] <= 180)).sum() / total_time * 100
time_below_70 = (cgm_data['CGM'] < 70).sum() / total_time * 100
time_below_54 = (cgm_data['CGM'] < 54).sum() / total_time * 100

# Adjust time_above_180 to exclude time_above_250
time_above_180_exclusive = time_above_180 - time_above_250

# Create a list with all the metrics
metrics = [
    num_days, percent_data_captured, mean_glucose, gmi, cv,
    time_above_250, time_above_180_exclusive, time_in_range,
    time_below_70, time_below_54
]

Metrics
```

Case 7

```
from datetime import datetime

# Convert 'Time' to datetime format
cgm_data['Time'] = pd.to_datetime(cgm_data['Time'])

# Calculate the number of days the sensor is active
num_days = (cgm_data['Time'].max() - cgm_data['Time'].min()).days + 1

# Assuming the sensor captures data every 5 minutes, calculate total possible readings
expected_readings_per_day = 24 * 60 / 5
total_expected_readings = expected_readings_per_day * num_days

# Calculate the percentage of sensor data captured
percent_captured = (len(cgm_data) / total_expected_readings) * 100

# Calculate mean glucose concentration
mean_glucose = cgm_data['CGM'].mean()

# Calculate Glucose Management Indicator (GMI)
# GMI formula: 3.31 + (0.02392 × mean glucose in mg/dL)
gmi = 3.31 + (0.02392 * mean_glucose)

# Calculate Glucose Variability (Coefficient of Variation)
cv_glucose = cgm_data['CGM'].std() / mean_glucose

# Calculate percentages for TAR, TIR, and TBR
tar_level_2 = (cgm_data[cgm_data['CGM'] > 250].shape[0] / len(cgm_data)) * 100
tar_level_1 = (cgm_data[cgm_data['CGM'] > 180].shape[0] / len(cgm_data)) * 100
tir = (cgm_data[(cgm_data['CGM'] >= 70) & (cgm_data['CGM'] <= 180)].shape[0] / len(cgm_data)) * 100
tbr_level_1 = (cgm_data[cgm_data['CGM'] < 70].shape[0] / len(cgm_data)) * 100
tbr_level_2 = (cgm_data[cgm_data['CGM'] < 54].shape[0] / len(cgm_data)) * 100

# Adjust TAR Level 1 to exclude TAR Level 2 readings for accurate TAR Level 1
tar_level_1_adjusted = tar_level_1 - tar_level_2

metrics = [
```

```
    num_days,
    percent_captured,
    mean_glucose,
    gmi,
    cv_glucose,
    tar_level_2,
    tar_level_1_adjusted,
    tir,
    tbr_level_1,
    tbr_level_2
]

Metrics
```

Case 8

```
from datetime import datetime

# Convert the 'Time' column to datetime format
cgm_data['Time'] = pd.to_datetime(cgm_data['Time'], format='%m/%d/%y %H:%M')

# Calculate the number of unique days the sensor is active
num_days = cgm_data['Time'].dt.date.nunique()

# Assuming the sensor should record a reading every 5 minutes, calculate the % of sensor data captured
expected_readings_per_day = 24 * 60 / 5
total_expected_readings = expected_readings_per_day * num_days
percent_captured = (len(cgm_data) / total_expected_readings) * 100

# Calculate the mean glucose concentration
mean_glucose = cgm_data['CGM'].mean()

# Calculate the Glucose Management Indicator (GMI)
# GMI formula: 3.31 + (0.02392 × mean glucose in mg/dL)
gmi = 3.31 + (0.02392 * mean_glucose)

# Calculate the glucose variability (coefficient of variation)
cv_glucose = (cgm_data['CGM'].std() / mean_glucose) * 100

# Calculate % times in various glucose ranges
total_time = len(cgm_data)
time_above_250 = (cgm_data['CGM'] > 250).sum() / total_time * 100
time_above_180 = (cgm_data['CGM'] > 180).sum() / total_time * 100
time_in_range = ((cgm_data['CGM'] >= 70) & (cgm_data['CGM'] <= 180)).sum() / total_time * 100
time_below_70 = (cgm_data['CGM'] < 70).sum() / total_time * 100
time_below_54 = (cgm_data['CGM'] < 54).sum() / total_time * 100

# Adjust the % time above range level 1 to exclude time counted in level 2
time_above_180_exclusive = time_above_180 - time_above_250

metrics = [
    num_days,  # Number of days the sensor is active
    percent_captured,  # % of sensor data captured
    mean_glucose,  # Mean glucose concentration
    gmi,  # Glucose management indicator
    cv_glucose,  # Glucose variability (coefficient of variation)
    time_above_250,  # % Time above TAR in level 2 (>250)
    time_above_180_exclusive,  # % Time above TAR in level 1 (>180) exclusive
    time_in_range,  # % Time in range (70-180mg/dL)
    time_below_70,  # % Time below TBR in level 1 (<70)
    time_below_54  # % Time below TBR in level 2 (<54)
]

metrics
```

Case 9

```
from datetime import datetime

# Convert the "Time" column to datetime format
cgm_data['Datetime'] = pd.to_datetime(cgm_data['Time'], format='%m/%d/%y %H:%M')

# Calculate the number of days the sensor is active
num_days = (cgm_data['Datetime'].max() - cgm_data['Datetime'].min()).days + 1

# Calculate % of sensor data captured
# Assuming the sensor records data every 5 minutes, there should be 288 readings per day
expected_readings_per_day = 288
total_expected_readings = num_days * expected_readings_per_day
percent_captured = (len(cgm_data) / total_expected_readings) * 100

# Calculate mean glucose concentration
mean_glucose = cgm_data['CGM'].mean()

# Calculate Glucose Management Indicator (GMI)
gmi = 3.31 + (0.02392 * mean_glucose)

# Calculate glucose variability (coefficient of variation)
coefficient_of_variation = cgm_data['CGM'].std() / mean_glucose
```

<table>
<tr><td></td><td>

```
# Calculate % time above target glucose range (TAR) in level 2 (>250)
time_above_250 = len(cgm_data[cgm_data['CGM'] > 250]) / len(cgm_data) * 100

# Calculate % time above target glucose range (TAR) in level 1 (>180)
time_above_180 = len(cgm_data[cgm_data['CGM'] > 180]) / len(cgm_data) * 100

# Calculate % time in range (70-180mg/dL)
time_in_range = len(cgm_data[(cgm_data['CGM'] >= 70) & (cgm_data['CGM'] <= 180)]) / len(cgm_data) * 100

# Calculate % time below target glucose range (TBR) in level 1 (<70)
time_below_70 = len(cgm_data[cgm_data['CGM'] < 70]) / len(cgm_data) * 100

# Calculate % time below target glucose range (TBR) in level 2 (<54)
time_below_54 = len(cgm_data[cgm_data['CGM'] < 54]) / len(cgm_data) * 100

# Create a list of the calculated metrics
metrics = [
    num_days, percent_captured, mean_glucose, gmi,
    coefficient_of_variation, time_above_250, time_above_180,
    time_in_range, time_below_70, time_below_54
]

Metrics
```

</td></tr>
<tr><td>Case 10</td><td>

```
from datetime import timedelta

# Parse the 'Time' column to datetime
cgm_data['Time'] = pd.to_datetime(cgm_data['Time'], format='%m/%d/%y %H:%M')

# Calculate the number of days the sensor is active
num_days = (cgm_data['Time'].max() - cgm_data['Time'].min()).days + 1

# Calculate % of sensor data captured (assuming the sensor should capture data every 5 minutes)
expected_measurements_per_day = 24 * 60 / 5
total_expected_measurements = expected_measurements_per_day * num_days
percent_data_captured = (len(cgm_data) / total_expected_measurements) * 100

# Calculate mean glucose concentration
mean_glucose = cgm_data['CGM'].mean()

# Glucose Management Indicator (GMI) calculation, using the formula: GMI = 3.31 + 0.02392 * mean glucose
gmi = 3.31 + 0.02392 * mean_glucose

# Glucose variability as the coefficient of variation (CV = standard deviation / mean * 100)
glucose_variability = (cgm_data['CGM'].std() / mean_glucose) * 100

# Calculate % Time in various ranges
time_in_tar_level2 = len(cgm_data[cgm_data['CGM'] > 250]) / len(cgm_data) * 100
time_in_tar_level1 = len(cgm_data[(cgm_data['CGM'] > 180) & (cgm_data['CGM'] <= 250)]) / len(cgm_data) * 100
time_in_range = len(cgm_data[(cgm_data['CGM'] >= 70) & (cgm_data['CGM'] <= 180)]) / len(cgm_data) * 100
time_in_tbr_level1 = len(cgm_data[cgm_data['CGM'] < 70]) / len(cgm_data) * 100
time_in_tbr_level2 = len(cgm_data[cgm_data['CGM'] < 54]) / len(cgm_data) * 100

# Time above target range (TAR) >180 includes both level 1 and level 2
time_above_tar = time_in_tar_level1 + time_in_tar_level2

# Compile the results into a list
results = [
    num_days,  # Number of days the sensor is active
    percent_data_captured,  # % of sensor data captured
    mean_glucose,  # Mean glucose concentration
    gmi,  # Glucose management indicator
    glucose_variability,  # Glucose variability (CV)
    time_in_tar_level2,  # % Time above target glucose range (TAR) in level 2 (>250)
    time_above_tar,  # % Time above target glucose range (TAR) in level 1 (>180) including level 2
    time_in_range,  # % Time in range (70-180mg/dL)
    time_in_tbr_level1,  # % Time below target glucose range (TBR) in level 1 (<70)
    time_in_tbr_level2  # % Time below target glucose range (TBR) in level 2 (<54)
]

Results
```

</td></tr>
</table>

**Table S8: Code written by GPT-4 for Quantitative Prompts**

## AGP Summarization Task Prompt Output

Responses generated on February 5th, 2024 using OpenAI Chat GPT Data Analyst Plugin with default parameters https://chat.openai.com/g/g-HMNcP6w7d-data-analyst .

| Case | GPT-4 Output (Recorded outputs only) |
|---|---|
| Case 1 | The CGM data provided covers 14 unique days, which meets the recommended period of analysis. Additionally, the CGM activity time is approximately 88.54%, which is above the recommended threshold of 70%.<br><br>Output Summary: This data is of good quality because it covers the recommended 14-day analysis period and has a CGM activity time well above the recommended 70% threshold.<br><br>—-----------------------------------------------------------<br><br>Hyperglycemia is a periodic concern for this patient, with a few days showing higher glucose levels. Notably, January 8th experienced the highest daily percentage of hyperglycemia (12.65%), followed by notable levels on January 13th and 18th. In terms of daily patterns, mornings and afternoons showed higher average glucose readings, with the morning being particularly high. The evening period registered the peak glucose level of 236 mg/dL. However, there were no instances of prolonged severe hyperglycemia, indicating that while there are periods of elevated glucose levels, they do not extend for more than an hour at a time. This information is valuable for identifying specific periods of the day or certain dates where glucose management may require closer attention or intervention.<br><br>—-----------------------------------------------------------<br><br>Hypoglycemia was not a prevalent issue for this patient. The few instances of hypoglycemia (glucose levels below 70 mg/dL) were observed on January 8th, 14th, and 20th, but these occurrences were minimal and did not constitute a significant part of the day. Notably, there were no instances of severe hypoglycemia (below 54 mg/dL) throughout the analysis period. In terms of daily patterns, a slight increase in hypoglycemic readings was noted during the late night and night, but these did not lead to prolonged events. Overall, the patient's glucose levels appear to be well-managed, with no prolonged hypoglycemia episodes, indicating effective control of blood sugar levels to avoid significant low glucose events.<br><br>—-----------------------------------------------------------<br><br>The analysis of glycemic variability showed that the patient's daily coefficient of variation (CV) consistently remained below the target of 36%, indicating good overall stability in glucose levels. The days with the highest CV, suggesting somewhat greater variability, were January 20th (27.04%), January 14th (27.06%), and January 18th (26.14%), although these values were still within acceptable limits. In terms of the interquartile range (IQR), the highest variability was observed during the evening (53.0 mg/dL) and night (47.0 mg/dL) times. This suggests that glucose levels fluctuate more during these periods compared to other times of the day. Identifying and addressing potential causes for these evening and night fluctuations could further improve the patient's glycemic control.<br><br>—-----------------------------------------------------------<br><br>Based on the analysis, the primary clinical concern appears to be intermittent hyperglycemia, particularly evident in the morning and afternoon periods, with the highest average glucose levels and occasional spikes above the hyperglycemic threshold. Although the instances of severe hyperglycemia were not prolonged or frequent, identifying and addressing the causes behind these higher glucose levels during specific times of the day could improve overall glycemic control. Hypoglycemia and glycemic variability do not present significant concerns, as the data indicates these aspects are generally well-managed within the recommended guidelines. |
| Case 2 | The analysis of the CGM data reveals:<br><br>The data covers 14 unique days, meeting the recommended period of analysis for 14 days.<br>The CGM activity time is approximately 88.81%, which is above the recommended threshold of 70%.<br>Output Summary: This CGM data is of good quality because it covers the recommended analysis period of 14 days and has a high CGM activity time of 88.81%, ensuring a comprehensive and reliable dataset for assessment.<br><br>—-----------------------------------------------------------<br><br>Hyperglycemia is a notable concern in this patient's CGM data. Interday analysis shows a high variability in glucose levels, with an average of nearly 50% of the day spent in hyperglycemia and peaks reaching as high as 386 mg/dL. Intraday trends reveal specific times of the day, particularly in the evening and night, where hyperglycemia is more pronounced. Critically, there are instances of prolonged severe hyperglycemia, with CGM readings exceeding 250 mg/dL for over an hour. For example, on January 8th, prolonged hyperglycemia was observed from 11:40 to 12:15 and again at 14:30 to 14:45. Similar patterns occurred on January 10th and 19th, indicating the need for targeted interventions during these specific times. These findings suggest the importance of reviewing treatment strategies and lifestyle factors that may influence these patterns.<br><br>—-----------------------------------------------------------<br><br>Hypoglycemia is not a predominant issue in this patient's CGM data. The analysis shows that the patient rarely spends time below the hypoglycemic threshold of 70 mg/dL, both on an interday and intraday basis. Severe hypoglycemia (below 54 mg/dL) was not observed in the data. There was only a single brief instance of prolonged hypoglycemia noted on January 17th between 00:10 and 00:20, but it did not reach the severe hypoglycemia threshold. Overall, the patient's glucose levels are well maintained above hypoglycemic levels throughout the majority of the monitoring period, indicating effective management of low glucose levels.<br><br>—-----------------------------------------------------------<br><br>The analysis of glycemic variability in the patient's data shows overall stability, with all daily coefficients of variation (CV) below the recommended threshold of 36%. The highest CV was observed on January 11th at 30.69%, while the lowest was on January 9th at 19.50%. Regarding the interquartile range (IQR), which reflects glucose fluctuations within specific time windows, the highest variability was noted during the night, with an IQR of approximately 119.75. In contrast, the early morning period exhibited the least variability, with an IQR around 45.00. These findings indicate that while daily glycemic variability is within acceptable limits, there are significant differences in glucose fluctuations across different times of the day, particularly between night and early morning.<br><br>—-----------------------------------------------------------<br><br>Based on the analysis and guidelines, the primary clinical concern for this patient is hyperglycemia. The data indicates significant periods of both hyperglycemia (glucose levels above 180 mg/dL) and severe hyperglycemia (levels above 250 mg/dL), particularly during certain times of the day and on specific dates. Although hypoglycemia and glycemic variability are not major issues, as evidenced by the minimal occurrences of hypoglycemia and the stable daily coefficients of variation, the consistent and prolonged instances of hyperglycemia warrant clinical attention. It suggests a need for reviewing and potentially adjusting the case's treatment regimen and lifestyle factors to better manage blood glucose levels. |
| Case 3 | The analysis reveals that:<br><br>The data covers 14 unique days, meeting the recommended period of analysis.<br>The percentage of CGM activity time is approximately 86.06%, which is above the recommended threshold of 70%.<br>Output Summary: This data is of good quality because it covers the recommended period of analysis (14 days) and has a CGM activity time greater than 70% (86.06%), indicating comprehensive monitoring within the period |

| | |
|---|---|
| | —-------------------------------------------------------------------------------<br><br>The analysis of the CGM data indicates that hyperglycemia, including instances of severe hyperglycemia, is a significant concern for this patient. Notably, January 10th emerged as a critical day with an average glucose level of 276.21 mg/dL, indicating extensive periods above the hyperglycemia threshold, including 83.97% of readings above 180 mg/dL and 47.39% above 250 mg/dL. Intraday trends revealed that hyperglycemia is particularly prevalent during the afternoon and night, with severe spikes noted on January 10th in the afternoon and January 17th at night. Prolonged hyperglycemia events were identified on several days, including a substantial episode on January 10th spanning from morning to afternoon, and multiple night-time events on January 9th, 17th, and 20th, suggesting challenges in managing glucose levels during these times. These findings highlight the need for targeted interventions to address periods of significant hyperglycemia, especially during identified problematic times of day.<br><br>—-------------------------------------------------------------------------------<br><br>The analysis of the CGM data for hypoglycemia shows that it is not a significant issue for this patient. A minor instance of hypoglycemia was observed on January 9th, where the glucose level briefly fell to 65 mg/dL during the afternoon, but this did not extend to a prolonged event or reach severe hypoglycemia levels. There were no instances of glucose levels falling below 54 mg/dL, and no prolonged periods of hypoglycemia exceeding 60 minutes were identified. This suggests that the patient's current management strategies for preventing hypoglycemia are generally effective, with only a singular, brief deviation noted. Monitoring should continue, but there appears to be no immediate cause for concern regarding hypoglycemia based on this data analysis.<br><br>—-------------------------------------------------------------------------------<br><br>The glycemic variability analysis revealed that several days exhibited high variation in glucose levels, notably on January 9th, 10th, 17th, and 20th, where the coefficient of variation exceeded the guideline of 36%. This indicates significant fluctuations in glucose levels on these days, suggesting that factors affecting glycemic control may need closer examination and management. Furthermore, the interquartile range (IQR) analysis identified the evening as the time of day with the highest glucose fluctuations, followed by the afternoon and night. The least variability was observed during the early morning hours. These findings highlight the need for targeted strategies to manage glucose levels more effectively, particularly during times of day identified with higher variability, to improve overall glycemic control for the patient.<br><br>—-------------------------------------------------------------------------------<br><br>Based on the analysis, the primary clinical concern is the significant hyperglycemia, particularly the instances of severe hyperglycemia and high glycemic variability. Days with notable hyperglycemia, such as January 10th and 17th, and times of day like the evening and afternoon, were identified as periods of high glucose levels and variability. The occurrence of prolonged hyperglycemia events further underscores the urgency of addressing these glycemic control issues. While hypoglycemia does not appear to be a significant problem for this patient, the high levels of variability and instances of severe hyperglycemia suggest that the current diabetes management strategy may need to be reevaluated and adjusted to better control blood glucose levels and minimize risks associated with hyperglycemia. |
| Case 4 | The CGM data analysis reveals the following:<br><br>The dataset covers 14 unique days, meeting the recommended period of analysis.<br>The CGM activity time is approximately 90.3%, which is above the recommended threshold of 70%.<br>Output Summary: This data is of good quality because it encompasses the recommended 14 days of analysis and has a CGM activity time well above the recommended threshold, ensuring a comprehensive and reliable dataset for further analysis.<br><br>—-------------------------------------------------------------------------------<br><br>The analysis of the CGM data indicates that hyperglycemia is a significant issue for the patient, with several days showing a high percentage of time spent above the hyperglycemia threshold. Notably, on January 9, 11, 15, 16, 17, 18, and 19, the patient spent a large part of the day in a hyperglycemic state, with January 15 showing an exceptionally high 98.6% of the time above the threshold. Severe hyperglycemia (>250 mg/dL) was also a concern, particularly on January 12 and 18, where more than half of the day was spent at these dangerous levels. Prolonged hyperglycemia events were frequent, with significant episodes occurring across various days, including multiple instances on January 9, 12, 15, 18, and 19 in different time windows such as the afternoon, morning, and late night. These findings suggest the need for a detailed review of the patient's treatment plan and lifestyle choices during these identified periods to address and mitigate hyperglycemia risks effectively.<br><br>—-------------------------------------------------------------------------------<br><br>The analysis of the CGM data for hypoglycemia reveals that low blood sugar levels are not a significant concern for the patient during the observed period. Only on January 10th was there a minor incidence of hypoglycemia (below 70 mg/dL), with a negligible portion of the day affected and no evidence of severe hypoglycemia (below 54 mg/dL) on any day. Furthermore, there were no instances of prolonged hypoglycemia, indicating that the patient did not experience extended periods of dangerously low glucose levels. This lack of significant hypoglycemia suggests that while managing high blood sugar levels is crucial for the patient, maintaining a balance to avoid overly aggressive lowering that could result in hypoglycemia is also being effectively achieved.<br><br>—-------------------------------------------------------------------------------<br><br>The glycemic variability analysis revealed that most days exhibited a coefficient of variation (CV) below the recommended threshold of 36%, indicating generally stable glucose levels. However, specific days, such as January 10, 12, and 18, demonstrated higher CVs, with January 18 approaching the threshold, suggesting notable fluctuations in glucose levels. The interquartile range (IQR) analysis highlighted January 12 and 18 as days with significantly larger fluctuations, indicative of pronounced glycemic variability. Furthermore, the analysis of IQR across different times of the day identified "Late night" and "Afternoon" as periods with the highest variability, pointing towards more substantial fluctuations during these times. This information could be crucial for tailoring patient management strategies, particularly focusing on dietary and activity adjustments during times of heightened variability to enhance glycemic control.<br><br>—-------------------------------------------------------------------------------<br><br>The primary clinical concern identified from the analysis is the significant issue of hyperglycemia, with several days showing a high percentage of time spent above the hyperglycemia threshold and instances of severe hyperglycemia. This is compounded by the observation of pronounced glycemic variability on certain days and at specific times, suggesting that while hypoglycemia is not a significant issue, the patient's glucose management strategy may need adjustment. The frequent and prolonged periods of high glucose levels, especially the severe hyperglycemia events, pose a considerable risk for diabetic complications. Therefore, addressing these hyperglycemic episodes and managing glycemic variability should be a priority in the patient's diabetes care plan. |
| Case 5 | The CGM data covers 14 unique days, meeting the recommended period of analysis. Additionally, the CGM activity time is approximately 97.67%, which is well above the recommended threshold of 70%.<br><br>Output Summary: The data is of good quality because it covers the recommended 14-day analysis period and has a high CGM activity time of approximately 97.67%, exceeding the recommended 70%.<br><br>—-------------------------------------------------------------------------------<br><br>The analysis of your CGM data indicates that hyperglycemia is not a significant concern. Interday trends show that on average, only about 0.63% of your time is spent in a hyperglycemic state (glucose > 180 mg/dL), with the highest daily percentage being around 5.21%. Intraday trends reveal that your average glucose levels vary throughout the day but do not frequently exceed the hyperglycemia threshold, with the highest recorded level being 208 mg/dL. Importantly, there were no instances of prolonged severe hyperglycemia (glucose levels > 250 mg/dL lasting over an hour) in the data. These findings suggest good overall glucose management, although it might be beneficial to review the days with slightly higher levels to understand any specific triggers or activities associated with these variations.<br><br>—-------------------------------------------------------------------------------<br><br>The analysis of your CGM data reveals that hypoglycemia is not a significant concern. Interday trends show minimal daily occurrences of hypoglycemia, with very low percentages of time spent below both the mild (below 70 mg/dL) and severe (below 54 mg/dL) hypoglycemic thresholds. Intraday trends do not indicate any consistent pattern or specific times of day when hypoglycemia is more likely to occur. Importantly, there were no instances of prolonged hypoglycemia, either mild or severe, on any of the days in the analyzed dataset. This suggests effective management of your glucose levels, with minimal risk of hypoglycemic episodes.<br><br>—------------------------------------------------------------------------------- |

| | |
|---|---|
| | The analysis of glycemic variability in your CGM data reveals excellent glucose control. No days were identified with a coefficient of variation exceeding the recommended 36%, indicating a consistent and stable management of glucose levels on a daily basis. Furthermore, the interquartile ranges for different times of the day – Morning, Afternoon, Evening, and Night – were consistently low, suggesting minimal variability in glucose levels throughout the day. This stability in glycemic control is a positive indicator of effective diabetes management strategies, and it minimizes the risk of complications associated with large glucose fluctuations.<br><br>—-------------------------------------------------------------<br><br>Based on the analysis of the CGM data and adherence to clinical guidelines, the primary clinical concern for the patient appears to be minimal. The patient's glucose levels are well-managed, with minimal instances of hyperglycemia or hypoglycemia. Glycemic variability is also within the recommended range, indicating stable glucose control. Therefore, the patient seems to be effectively managing their diabetes, and there are no significant clinical concerns identified from this data analysis. However, continued monitoring and routine check-ups are advisable to maintain this level of control. |
| Case 6 | Duration of Data Collection: The dataset contains CGM readings for exactly 14 unique days, meeting the recommended period of analysis for assessing diabetes management.<br><br>CGM Activity Time: The dataset has a CGM activity time of approximately 92.4%, which is significantly above the recommended threshold of 70%.<br><br>Output Summary<br>The data is of good quality because it covers the recommended 14-day analysis period and has a high CGM activity time of over 92%, ensuring a comprehensive overview of the patient's glucose levels.<br><br>—-------------------------------------------------------------<br><br>Hyperglycemia is a significant concern for this patient, with both frequent and severe instances noted. Interday analysis revealed particularly high glucose levels on specific days, such as January 21 and 13, where over 70% of readings were above the hyperglycemia threshold. Intraday trends showed the highest glucose peaks during late night and night, indicating a pattern of elevated glucose levels during these periods. Prolonged hyperglycemic events, where glucose levels were above 250 mg/dL for an hour or more, were observed on several days, including the afternoon and night of January 9, and the early morning and morning of January 10. These findings highlight the need for a focused review of the patient's diabetes management, especially during the identified times and days.<br><br>—-------------------------------------------------------------<br><br>Hypoglycemia is generally not a major concern for this patient, with only a few isolated instances. The most notable occurrence was on January 20, 2023, when 6.6% of the day's readings were below the hypoglycemia threshold of 70 mg/dL, particularly during late night. This specific instance indicates a need for cautious nighttime glucose management. Intraday analysis revealed that the lowest glucose readings typically occurred during the late-night hours, though severe hypoglycemia (below 54 mg/dL) was not a prominent issue. There was one prolonged hypoglycemic event on January 20, highlighting the importance of monitoring and possibly adjusting treatment strategies during late-night periods. Overall, while hypoglycemia is not widespread, vigilance during late-night hours is advisable.<br><br>—-------------------------------------------------------------<br><br>"The analysis of glycemic variability within the dataset revealed significant fluctuations in glucose levels on specific days and during certain times of the day. Notably, January 18 and January 9 exhibited high coefficients of variation, 44.54% and 42.41% respectively, surpassing the recommended threshold of 36%. This indicates considerable daily variability in glucose levels on these days, suggesting the need for closer monitoring or adjustments in diabetes management. Regarding the interquartile range, the most significant variability in glucose readings occurred during late night (IQR of 112) and evening (IQR of 102), indicating these periods as the times with the highest fluctuations. These findings underscore the importance of focusing on stabilizing glucose levels, particularly during late nights and evenings, to manage glycemic variability effectively.<br><br>—-------------------------------------------------------------<br><br>Based on the analysis and guidelines, the primary clinical concern for this patient is the significant hyperglycemia, including severe hyperglycemia, with frequent high glucose levels and substantial daily variability. Particular attention should be given to managing elevated glucose levels during late night and evening hours, as well as on days like January 18 and 9, which showed high glycemic variability. Although hypoglycemia is not a widespread issue, occasional monitoring, especially during late-night hours, is advisable. The focus should be on stabilizing blood glucose levels to avoid extreme highs and manage variability effectively. |
| Case 7 | Based on the analysis:<br><br>The CGM data covers 14 unique days, which meets the recommended period of analysis for 14 days.<br>The CGM was active approximately 95.49% of the time, well above the recommended activity time of 70%.<br>Output Summary: The data is of good quality as it covers the recommended 14 days and has a high CGM activity time of about 95.49%.<br><br>—-------------------------------------------------------------<br><br>Hyperglycemia is a significant issue for this patient, with several days showing elevated glucose levels. Notably, January 9th and 18th were particularly concerning, with extremely high percentages of the day spent in severe hyperglycemia. Intraday trends indicate that morning glucose levels are consistently high, followed by elevated levels in the afternoon and evening. Prolonged severe hyperglycemia events were frequent; for instance, on January 8th, these events spanned across the afternoon, evening, morning, and night. Such patterns suggest the need for careful review and potential adjustments in diabetes management, especially considering the severity and frequency of these hyperglycemic episodes.<br><br>—-------------------------------------------------------------<br><br>Hypoglycemia is generally not a significant concern for this patient, as the data shows very few instances of low glucose levels. The only notable exception occurred on January 16th during late night, where both hypoglycemia (below 70 mg/dL) and severe hypoglycemia (below 54 mg/dL) were observed for a short duration. This isolated event of prolonged hypoglycemia, lasting over 60 minutes for hypoglycemia and over 15 minutes for severe hypoglycemia, warrants attention to understand possible triggers or specific conditions on that night. Otherwise, the patient's glucose levels were mostly stable and above the hypoglycemic thresholds.<br><br>—-------------------------------------------------------------<br><br>Output Summary: The glycemic variability analysis reveals that most days exhibit stable glucose levels with a Coefficient of Variation (CV) below the recommended 36%. However, increased variability was noted on January 13th, 15th, 16th, and particularly on January 21st, where the CV exceeded the threshold, indicating more fluctuating glucose levels. The Daily Interquartile Range (IQR) analysis showed that mornings and nights are periods with the highest glucose variability, with mornings having an average IQR around 51.7 and nights around 59.1. These findings suggest that while the patient's overall glycemic control is relatively stable, there are specific times, particularly mornings and nights, where glucose levels demonstrate more significant fluctuations.<br><br>—-------------------------------------------------------------<br><br>Based on the analysis, the primary clinical concern for this patient is the frequent and severe hyperglycemia. Several days, notably January 9th and 18th, showed extremely high levels of glucose and a significant portion of time spent in severe hyperglycemia. Additionally, the patient experienced substantial glucose variability on certain days and during specific times (especially mornings and nights), which could complicate diabetes management. While hypoglycemia does not appear to be a major issue, the high and variable hyperglycemic episodes are a significant concern and should be addressed with the patient's healthcare provider to optimize their diabetes treatment plan. |
| Case 8 | The data covers 14 unique days and has a CGM activity time of approximately 91.5%.<br><br>Output Summary:<br>This data is of good quality because it meets the recommended analysis period of 14 days and has a CGM activity time greater than 70%, specifically around 91.5%.<br><br>—-------------------------------------------------------------<br><br>Hyperglycemia is a noticeable concern for this patient, with evidence of glucose levels exceeding the hyperglycemia threshold of 180 mg/dL on several days, though no instances of severe hyperglycemia (over 250 mg/dL) were recorded on a daily aggregate level. The data did not show any prolonged severe hyperglycemia events, indicating that while |

| | |
|---|---|
| | glucose levels frequently rise above desired levels, they do not remain at critically high levels for extended periods. Intraday analysis revealed varying glucose levels throughout the day, suggesting that certain times may be more prone to hyperglycemic episodes; however, without severe spikes reaching the threshold for prolonged concern. This information can be useful for identifying patterns in daily activities or dietary habits that may influence glucose levels. It is important for the patient to be aware of these trends to manage and mitigate hyperglycemic episodes more effectively.<br><br>—--------------------------------------------------------------------------------<br><br>The analysis of the CGM data for hypoglycemia indicates that this patient does not experience significant issues with low blood sugar levels. No days were identified with a concerning number of readings below the hypoglycemia or severe hypoglycemia thresholds, suggesting that episodes of low glucose are not dominating the patient's glucose management. Furthermore, there were no instances of prolonged hypoglycemia, where glucose levels stayed dangerously low for extended periods. This lack of prolonged hypoglycemic events is a positive sign, indicating effective management of glucose levels to avoid the risks associated with low blood sugar. It suggests that the patient's current diabetes management plan is effectively preventing severe hypoglycemia, a crucial goal in the treatment of diabetes.<br><br>—--------------------------------------------------------------------------------<br><br>The analysis of glycemic variability within the CGM data revealed no days with a coefficient of variation exceeding 36%, indicating that the patient maintains relatively stable glucose levels on a daily basis without significant day-to-day fluctuations. This stability suggests effective management of glycemic variability, a crucial factor in minimizing the risk of diabetes-related complications. The interquartile range (IQR), which measures the spread of the middle 50% of glucose readings, varied across different times of the day, with a mean IQR of approximately 26.75 mg/dL. Although there are some fluctuations in glucose levels throughout the day, these did not show extreme variability, indicating controlled glucose levels during different times. This analysis underscores the importance of ongoing monitoring and management to maintain this level of glycemic control and stability.<br><br>—--------------------------------------------------------------------------------<br><br>Based on the analysis, the primary clinical concern appears to be hyperglycemia, with glucose levels frequently exceeding the recommended upper limit of 180 mg/dL, though not reaching severe hyperglycemia levels above 250 mg/dL for prolonged periods. While there are no significant concerns regarding hypoglycemia or excessive glycemic variability, managing and reducing episodes of hyperglycemia remains a crucial aspect of the patient's diabetes management. Identifying and addressing the causes of these hyperglycemic episodes, whether related to diet, physical activity, or insulin therapy adjustments, should be a focus to minimize the risk of diabetes-related complications. |
| Case 9 | The dataset contains data for 14 unique days, and the CGM was active for approximately 99.16% of the time.<br><br>Given this information, we can conclude that the data is of good quality because:<br><br>It covers the recommended period of analysis of 14 days.<br>The CGM activity time is well above the recommended threshold of 70%.<br><br>Output Summary: This CGM data is of good quality because it spans over the recommended 14 unique days and has a high activity time of approximately 99.16%, indicating comprehensive monitoring.<br><br>—--------------------------------------------------------------------------------<br><br>The analysis of the CGM data reveals significant concerns regarding hyperglycemia for this patient, particularly on certain days and during specific times. Notably, January 12th and 13th stood out with exceptionally high average glucose levels and significant portions of the day spent above the hyperglycemic threshold, indicating severe hyperglycemia. Additionally, there were multiple instances of prolonged severe hyperglycemia, notably during the late night and early morning of January 12th, and during the afternoon and evening of January 13th. These patterns suggest that the patient's glucose levels tend to rise significantly during the late night to early morning and in the afternoon to evening. Identifying specific behaviors or circumstances on these days and times, such as diet, exercise, or medication management, could be crucial in developing strategies to mitigate these hyperglycemic events.<br><br>—--------------------------------------------------------------------------------<br><br>The hypoglycemia analysis indicates that while hypoglycemic events were not widespread across all days, January 11th emerged as a significant concern due to notable levels of hypoglycemia, including both below 70 mg/dL for a substantial part of the evening and severe hypoglycemia below 54 mg/dL. Additionally, prolonged hypoglycemia events were observed on January 11th and 12th, particularly in the evening and night, suggesting a pattern where the risk of hypoglycemia increases during these times. These findings highlight the importance of closely monitoring glucose levels and possibly adjusting treatment or behaviors around these identified times to mitigate the risk of future hypoglycemic episodes.<br><br>—--------------------------------------------------------------------------------<br><br>The glycemic variability analysis highlighted significant fluctuations in glucose control, particularly on January 11th, 12th, and 13th, where the coefficient of variation exceeded the target of less than 36%, indicating high glycemic variability. These days, especially with CVs of 43.86%, 55.17%, and 53.41% respectively, suggest inconsistent glucose management and potential risk areas for the patient. Additionally, the evening time window exhibited the highest interquartile range (IQR) of glucose readings, pointing to a broader variability in glucose levels during this period. This suggests that the patient's glucose levels are more unstable in the evenings, possibly due to dinner meals, activity levels, or the timing of medication. Addressing these specific times and patterns could be key to achieving more stable glucose control.<br><br>—--------------------------------------------------------------------------------<br><br>The primary clinical concern based on the analysis is the significant glycemic variability, particularly the instances of severe hyperglycemia and the days with high coefficient of variation, indicating inconsistent glucose control. The days with prolonged severe hyperglycemia (glucose levels above 250 mg/dL for extended periods) and the evenings with high interquartile ranges of glucose readings highlight the need for closer monitoring and possibly adjustments in the management plan. These findings suggest that the patient may benefit from tailored interventions to address both hyperglycemia and glycemic variability, potentially involving changes in insulin therapy, dietary adjustments, and lifestyle modifications to stabilize glucose levels and reduce the risk of complications. |
| Case 10 | The CGM data covers 14 unique days with a span of 14 days, and the CGM activity time is approximately 97.37%. This means the dataset meets both recommended criteria for analysis:<br><br>It has 14 unique days of data, aligning with the recommended period of analysis.<br>The CGM was active about 97.37% of the time, which is well above the recommended threshold of 70%.<br>Output Summary: The data is of good quality because it covers the recommended period of 14 days and has a high CGM activity time of approximately 97.37%, ensuring a comprehensive analysis<br><br>—--------------------------------------------------------------------------------<br><br>The analysis of the CGM data indicates that hyperglycemia is a concern for this patient, particularly with several instances of severe hyperglycemia. Notably, the evenings of January 11th and the afternoons of January 12th and January 18th exhibited significant hyperglycemia, with glucose levels peaking at 285 mg/dL, 271 mg/dL, and 332 mg/dL, respectively. These findings suggest a pattern of postprandial hyperglycemia that might be linked to meal composition or timing. Prolonged periods of severe hyperglycemia, lasting for an hour or more, were identified on January 8th (early hours), January 11th (late afternoon and early evening), January 12th (early afternoon), and January 18th (midday and early afternoon), indicating critical times when the patient's glucose management was inadequate. These specific instances provide actionable insights for the patient and their healthcare provider to explore potential triggers and adjust treatment plans accordingly.<br><br>—--------------------------------------------------------------------------------<br><br>The analysis of the CGM data reveals that hypoglycemia is a notable concern for this patient, particularly with occurrences of prolonged and severe hypoglycemic events. Notably, the nights transitioning from January 11th to 12th and the early morning of January 16th experienced significant periods of hypoglycemia below 70 mg/dL for more than an hour, and instances where glucose levels dropped below 54 mg/dL for more than 15 minutes. These findings suggest a pattern of nocturnal hypoglycemia, which could be related to insulin dosing, meal planning, or physical activity levels. Addressing these trends is crucial for preventing hypoglycemia and ensuring the patient's safety, indicating a need for potential adjustments in the patient's diabetes management strategy, especially in terms of nighttime insulin dosing or snack recommendations. |

| | |
|---|---|
| | —---------------------------------------------------------------------------------------------------------------------------------------<br><br>The analysis of glycemic variability in the CGM dataset reveals an overall stable pattern, with an average daily coefficient of variation (CV) at 24.10%, indicating good glycemic control for most days. However, there was a day where the CV spiked to 43.08%, highlighting a day with significantly higher glycemic variability, which may warrant closer examination to understand potential causes such as dietary choices, activity levels, or insulin dosing. The interquartile range (IQR) analysis shows higher variability during the night, early morning, and afternoon times, with the night time window exhibiting the highest IQR at 50.00 mg/dL. These findings suggest that these periods are particularly prone to fluctuations in blood glucose levels, and targeted strategies may be needed to address this variability and enhance the patient's glycemic stability.<br><br>—---------------------------------------------------------------------------------------------------------------------------------------<br><br>The primary clinical concern based on the analysis is the presence of both significant hyperglycemia and hypoglycemia events, indicating challenges in maintaining stable glycemic control. Specifically, the occurrence of severe hyperglycemia, with glucose readings frequently exceeding 250 mg/dL, and instances of prolonged hypoglycemia below 70 mg/dL, suggest risks for both acute and long-term complications. Additionally, the variability in glucose levels, especially during specific times of the day, points to the need for adjustments in the patient's diabetes management plan. Addressing these fluctuations through tailored interventions could help mitigate risks and improve overall glycemic stability. |

**Table S9: Qualitative Prompts Output**

## AGP Summarization Task Prompt Code Written

| Case | Tasks | Code Generated |
|---|---|---|
| Case 1 | Data Quality | (code below) |

```
# Extracting the date and time from the 'Time' column
cgm_data['DateTime'] = pd.to_datetime(cgm_data['Time'])

# Checking the date range of the dataset
date_range = cgm_data['DateTime'].dt.date.unique()

# Counting the number of unique days in the dataset
unique_days = len(date_range)

# Checking the total possible readings and actual readings
# Assuming readings are taken every 5 minutes, there are 288 readings per day (24*60/5)
total_possible_readings = unique_days * 288
actual_readings = cgm_data.shape[0]

# Calculating the percentage of CGM activity time
cgm_activity_percentage = (actual_readings / total_possible_readings) * 100

unique_days, cgm_activity_percentage
```

| Case | Tasks | Code Generated |
|---|---|---|
| | Hyperglycemia | (code below) |

```
import numpy as np

# Defining hyperglycemia thresholds
hyperglycemia_threshold = 180
severe_hyperglycemia_threshold = 250

# Extracting date and time information for analysis
cgm_data['Date'] = cgm_data['DateTime'].dt.date
cgm_data['Hour'] = cgm_data['DateTime'].dt.hour

# Breaking the day into 4-hour time windows and labeling them
time_windows = {0: "Late night", 4: "Early morning", 8: "Morning", 12: "Afternoon", 16: "Evening", 20:
"Night"}
cgm_data['TimeWindow'] = pd.cut(cgm_data['Hour'], bins=list(time_windows.keys()) + [24],
                                labels=list(time_windows.values()), right=False, include_lowest=True)

# 1. Interday trends: Identifying days with high levels of glucose or high percentage of the day above
hyperglycemic thresholds
daily_hyperglycemia = cgm_data[cgm_data['CGM'] > hyperglycemia_threshold].groupby('Date').size()
daily_severe_hyperglycemia = cgm_data[cgm_data['CGM'] >
severe_hyperglycemia_threshold].groupby('Date').size()
total_daily_readings = cgm_data.groupby('Date').size()
percentage_daily_hyperglycemia = (daily_hyperglycemia / total_daily_readings) * 100
percentage_daily_severe_hyperglycemia = (daily_severe_hyperglycemia / total_daily_readings) * 100

# 2. Intraday trends: Maximum and average glucose in each time window
intraday_trends = cgm_data.groupby('TimeWindow').agg(MaxGlucose=('CGM', 'max'), AvgGlucose=('CGM', 'mean'))

# 3. Prolonged hyperglycemia: Identifying periods of severe hyperglycemia lasting for an hour or more
cgm_data['SevereHyperglycemia'] = cgm_data['CGM'] > severe_hyperglycemia_threshold
prolonged_hyperglycemia = cgm_data.groupby(['Date', 'TimeWindow'])['SevereHyperglycemia'].rolling(window=12,
min_periods=12).sum()
prolonged_hyperglycemia = prolonged_hyperglycemia.reset_index().rename(columns={'SevereHyperglycemia':
'Count'})
prolonged_events = prolonged_hyperglycemia[prolonged_hyperglycemia['Count'] >= 12]

# Preparing results
interday_trends = pd.concat([percentage_daily_hyperglycemia, percentage_daily_severe_hyperglycemia], axis=1)
interday_trends.columns = ['% Hyperglycemia', '% Severe Hyperglycemia']
```

```
prolonged_events_summary = prolonged_events.groupby(['Date', 'TimeWindow']).size()

interday_trends, intraday_trends, prolonged_events_summary
```

**Hypoglycemia**

```
# Defining hypoglycemia thresholds
hypoglycemia_threshold = 70
severe_hypoglycemia_threshold = 54

# 1. Interday trends: Identifying days with low levels of glucose or high percentage of the day below
hypoglycemic thresholds
daily_hypoglycemia = cgm_data[cgm_data['CGM'] < hypoglycemia_threshold].groupby('Date').size()
daily_severe_hypoglycemia = cgm_data[cgm_data['CGM'] < severe_hypoglycemia_threshold].groupby('Date').size()
percentage_daily_hypoglycemia = (daily_hypoglycemia / total_daily_readings) * 100
percentage_daily_severe_hypoglycemia = (daily_severe_hypoglycemia / total_daily_readings) * 100

# 2. Intraday trends: Identifying if hypoglycemia occurs at certain times of the day
intraday_hypoglycemia_trends = cgm_data[cgm_data['CGM'] <
hypoglycemia_threshold].groupby('TimeWindow').size()
intraday_severe_hypoglycemia_trends = cgm_data[cgm_data['CGM'] <
severe_hypoglycemia_threshold].groupby('TimeWindow').size()

# 3. Prolonged hypoglycemia: Identifying periods of hypoglycemia lasting for 60 minutes or more, and severe
hypoglycemia for 15 minutes or more
cgm_data['Hypoglycemia'] = cgm_data['CGM'] < hypoglycemia_threshold
cgm_data['SevereHypoglycemia'] = cgm_data['CGM'] < severe_hypoglycemia_threshold
prolonged_hypoglycemia = cgm_data.groupby(['Date', 'TimeWindow'])['Hypoglycemia'].rolling(window=12,
min_periods=12).sum()
prolonged_severe_hypoglycemia = cgm_data.groupby(['Date',
'TimeWindow'])['SevereHypoglycemia'].rolling(window=3, min_periods=3).sum()
prolonged_hypoglycemia = prolonged_hypoglycemia.reset_index().rename(columns={'Hypoglycemia': 'Count'})
prolonged_severe_hypoglycemia =
prolonged_severe_hypoglycemia.reset_index().rename(columns={'SevereHypoglycemia': 'Count'})
prolonged_hypo_events = prolonged_hypoglycemia[prolonged_hypoglycemia['Count'] >= 12]
prolonged_severe_hypo_events = prolonged_severe_hypoglycemia[prolonged_severe_hypoglycemia['Count'] >= 3]

# Preparing results
interday_hypo_trends = pd.concat([percentage_daily_hypoglycemia, percentage_daily_severe_hypoglycemia],
axis=1)
interday_hypo_trends.columns = ['% Hypoglycemia', '% Severe Hypoglycemia']
prolonged_hypo_summary = prolonged_hypo_events.groupby(['Date', 'TimeWindow']).size()
prolonged_severe_hypo_summary = prolonged_severe_hypo_events.groupby(['Date', 'TimeWindow']).size()

interday_hypo_trends, intraday_hypoglycemia_trends, intraday_severe_hypoglycemia_trends,
prolonged_hypo_summary, prolonged_severe_hypo_summary
```

**Glycemic Variability**

```
# Calculating the Coefficient of Variation (CV) for each day
daily_cgm_stats = cgm_data.groupby('Date')['CGM'].agg(['mean', 'std'])
daily_cgm_stats['CoefficientOfVariation'] = (daily_cgm_stats['std'] / daily_cgm_stats['mean']) * 100

# Calculating the Interquartile Range (IQR) for each time window
time_window_cgm_stats = cgm_data.groupby('TimeWindow')['CGM'].agg(['quantile'])
time_window_cgm_stats['IQR'] = time_window_cgm_stats['quantile'].apply(lambda x: x(0.75) - x(0.25))

daily_cgm_stats, time_window_cgm_stats['IQR']

*** [ERROR] ***

# Correcting the calculation of Interquartile Range (IQR) for each time window
time_window_cgm_stats = cgm_data.groupby('TimeWindow')['CGM'].agg(['quantile'])
time_window_cgm_stats['IQR'] = cgm_data.groupby('TimeWindow')['CGM'].quantile(0.75) -
cgm_data.groupby('TimeWindow')['CGM'].quantile(0.25)

daily_cgm_stats, time_window_cgm_stats['IQR']
```

**Case 2** — **Data Quality**

```
from datetime import datetime

# Convert the 'Time' column to datetime format for easier processing
cgm_data['Time'] = pd.to_datetime(cgm_data['Time'])

# Calculate the number of unique days
unique_days = cgm_data['Time'].dt.date.nunique()

# Assuming the CGM records data every 5 minutes, calculate the expected number of readings per day
expected_readings_per_day = 24 * 60 / 5  # 288 readings per day

# Total expected readings for the unique days
total_expected_readings = unique_days * expected_readings_per_day

# Actual number of readings
```

```
actual_readings = len(cgm_data)

# Calculate the percentage of CGM activity time
cgm_activity_percentage = (actual_readings / total_expected_readings) * 100

unique_days, cgm_activity_percentage
```

Hyperglycemia

```
# Define hyperglycemia thresholds
hyperglycemia_threshold = 180
severe_hyperglycemia_threshold = 250

# Add a column to the dataframe indicating if each reading is hyperglycemic or severely hyperglycemic
cgm_data['Hyperglycemic'] = cgm_data['CGM'] > hyperglycemia_threshold
cgm_data['Severely_Hyperglycemic'] = cgm_data['CGM'] > severe_hyperglycemia_threshold

# Group the data by date to analyze interday trends
interday_analysis = cgm_data.groupby(cgm_data['Time'].dt.date).agg({
    'Hyperglycemic': 'mean',
    'Severely_Hyperglycemic': 'mean',
    'CGM': ['max', 'mean']
})

# Define the 4-hour time windows for intraday analysis
time_windows = [
    ("Late night", 0, 4),
    ("Early morning", 4, 8),
    ("Morning", 8, 12),
    ("Afternoon", 12, 16),
    ("Evening", 16, 20),
    ("Night", 20, 24)
]

# Function to assign each time to a time window
def assign_time_window(hour):
    for window_name, start_hour, end_hour in time_windows:
        if start_hour <= hour < end_hour:
            return window_name
    return "Unknown"

# Add a column for time window
cgm_data['Time_Window'] = cgm_data['Time'].dt.hour.apply(assign_time_window)

# Group the data by time window for intraday analysis
intraday_analysis = cgm_data.groupby('Time_Window').agg({
    'CGM': ['max', 'mean'],
    'Hyperglycemic': 'mean',
    'Severely_Hyperglycemic': 'mean'
}).sort_values(by=('CGM', 'mean'), ascending=False)

# Prolonged hyperglycemia: check for periods where glucose readings are over 250 for an hour or more
cgm_data['Severe_Hyperglycemia_Continuous'] = cgm_data['Severely_Hyperglycemic'].rolling(12).sum() == 12
prolonged_hyperglycemia = cgm_data[cgm_data['Severe_Hyperglycemia_Continuous']]

# Summarize the findings
interday_summary = interday_analysis.describe()
intraday_summary = intraday_analysis.describe()
prolonged_hyperglycemia_summary = prolonged_hyperglycemia['Time'].dt.strftime('%Y-%m-%d %H:%M').unique()

interday_summary, intraday_summary, prolonged_hyperglycemia_summary
```

Hypoglycemia

```
# Define hypoglycemia thresholds
hypoglycemia_threshold = 70
severe_hypoglycemia_threshold = 54

# Add columns indicating if each reading is hypoglycemic or severely hypoglycemic
cgm_data['Hypoglycemic'] = cgm_data['CGM'] < hypoglycemia_threshold
cgm_data['Severely_Hypoglycemic'] = cgm_data['CGM'] < severe_hypoglycemia_threshold

# Group the data by date for interday analysis
interday_hypo_analysis = cgm_data.groupby(cgm_data['Time'].dt.date).agg({
    'Hypoglycemic': 'mean',
    'Severely_Hypoglycemic': 'mean',
    'CGM': ['min', 'mean']
})

# Group the data by time window for intraday analysis
intraday_hypo_analysis = cgm_data.groupby('Time_Window').agg({
    'CGM': ['min', 'mean'],
    'Hypoglycemic': 'mean',
    'Severely_Hypoglycemic': 'mean'
}).sort_values(by=('CGM', 'mean'))

# Prolonged hypoglycemia: check for periods where glucose readings are below 70 for more than 60 minutes, or
below 54 for more than 15 minutes
cgm_data['Prolonged_Hypoglycemia_70'] = cgm_data['Hypoglycemic'].rolling(12).sum() >= 12
```

| | | |
|---|---|---|
| | | cgm_data['Prolonged_Hypoglycemia_54'] = cgm_data['Severely_Hypoglycemic'].rolling(3).sum() >= 3<br>prolonged_hypoglycemia_70 = cgm_data[cgm_data['Prolonged_Hypoglycemia_70']]<br>prolonged_hypoglycemia_54 = cgm_data[cgm_data['Prolonged_Hypoglycemia_54']]<br><br># Summarize the findings<br>interday_hypo_summary = interday_hypo_analysis.describe()<br>intraday_hypo_summary = intraday_hypo_analysis.describe()<br>prolonged_hypoglycemia_70_summary = prolonged_hypoglycemia_70['Time'].dt.strftime('%Y-%m-%d %H:%M').unique()<br>prolonged_hypoglycemia_54_summary = prolonged_hypoglycemia_54['Time'].dt.strftime('%Y-%m-%d %H:%M').unique()<br><br>interday_hypo_summary, intraday_hypo_summary, prolonged_hypoglycemia_70_summary,<br>prolonged_hypoglycemia_54_summary |
| | Glycemic Variability | # Calculating the coefficient of variation for each day<br>cgm_data['Date'] = cgm_data['Time'].dt.date<br>daily_cv = cgm_data.groupby('Date')['CGM'].agg(['mean', 'std'])<br>daily_cv['Coefficient_of_Variation'] = (daily_cv['std'] / daily_cv['mean']) * 100<br><br># Calculate the interquartile range (IQR) for each time window<br>intraday_iqr = cgm_data.groupby('Time_Window')['CGM'].agg(['25%', '75%'])<br>intraday_iqr['IQR'] = intraday_iqr['75%'] - intraday_iqr['25%']<br><br># Summarize the findings<br>daily_cv_summary = daily_cv.describe()<br>intraday_iqr_summary = intraday_iqr.describe()<br><br>daily_cv_summary, intraday_iqr_summary, daily_cv, intraday_iqr<br><br>**** [ERROR] ***<br><br># Correcting the calculation of the interquartile range (IQR) for each time window<br>intraday_iqr = cgm_data.groupby('Time_Window')['CGM'].agg(['quantile']).unstack().T<br>intraday_iqr.columns = ['25%', '75%']<br>intraday_iqr['IQR'] = intraday_iqr['75%'] - intraday_iqr['25%']<br><br># Recalculate the summaries<br>daily_cv_summary = daily_cv.describe()<br>intraday_iqr_summary = intraday_iqr.describe()<br><br>daily_cv_summary, intraday_iqr_summary, daily_cv, intraday_iqr<br><br>*** [ERROR] ***<br><br># Correcting the method to calculate the interquartile range (IQR) for each time window<br>intraday_iqr = cgm_data.groupby('Time_Window')['CGM'].agg(lambda x: x.quantile(0.75) - x.quantile(0.25))<br><br># Recalculating the summaries<br>daily_cv_summary = daily_cv.describe()<br>intraday_iqr_summary = intraday_iqr.describe()<br><br>daily_cv_summary, intraday_iqr_summary, daily_cv, intraday_iqr |
| | | |
| Case 3 | Data Quality | # Convert the 'Time' column to datetime format to facilitate analysis<br>cgm_data['DateTime'] = pd.to_datetime(cgm_data['Time'], format='%m/%d/%y %H:%M')<br><br># Calculate the number of unique days<br>unique_days = cgm_data['DateTime'].dt.date.nunique()<br><br># Calculate the total possible readings (assuming continuous data collection every 5 minutes for 14 days)<br>total_possible_readings = 14 * 24 * 60 / 5<br><br># Calculate the actual number of readings provided<br>actual_readings = cgm_data.shape[0]<br><br># Calculate the percentage of CGM activity time<br>percentage_cgm_activity_time = (actual_readings / total_possible_readings) * 100<br><br>unique_days, percentage_cgm_activity_time |
| | Hyperglycemia | # Define hyperglycemia thresholds<br>hyperglycemia_threshold = 180<br>severe_hyperglycemia_threshold = 250<br><br># Create a column for date without time to help with daily analysis<br>cgm_data['Date'] = cgm_data['DateTime'].dt.date<br><br># Group the data by Date and calculate the percentage of readings above the hyperglycemia thresholds<br>daily_hyperglycemia = cgm_data.groupby('Date').apply( |

| | | |
|---|---|---|
| | | <pre>    lambda x: pd.Series({<br>        'Percent_above_180': (x['CGM'] > hyperglycemia_threshold).mean() * 100,<br>        'Percent_above_250': (x['CGM'] > severe_hyperglycemia_threshold).mean() * 100,<br>        'Max_CGM': x['CGM'].max(),<br>        'Average_CGM': x['CGM'].mean()<br>    })<br>).reset_index()<br><br># Find days with notable hyperglycemia<br>notable_hyperglycemia_days = daily_hyperglycemia[(daily_hyperglycemia['Percent_above_180'] > 0) \|<br>                                                 (daily_hyperglycemia['Percent_above_250'] > 0)]<br><br>notable_hyperglycemia_days<br><br>*** [PAUSE] ***<br><br># Map the hours of the day to the specified time windows<br>def map_to_time_window(hour):<br>    if 0 <= hour < 4:<br>        return "Late night"<br>    elif 4 <= hour < 8:<br>        return "Early morning"<br>    elif 8 <= hour < 12:<br>        return "Morning"<br>    elif 12 <= hour < 16:<br>        return "Afternoon"<br>    elif 16 <= hour < 20:<br>        return "Evening"<br>    else:  # 20 <= hour < 24<br>        return "Night"<br><br># Apply the mapping to the data<br>cgm_data['TimeWindow'] = cgm_data['DateTime'].dt.hour.apply(map_to_time_window)<br><br># Calculate the average and maximum CGM for each time window across all days<br>intraday_hyperglycemia = cgm_data.groupby(['Date', 'TimeWindow']).agg(<br>    Average_CGM=('CGM', 'mean'),<br>    Max_CGM=('CGM', 'max')<br>).reset_index()<br><br># Identify time windows with hyperglycemia<br>intraday_hyperglycemia_max = intraday_hyperglycemia[intraday_hyperglycemia['Max_CGM'] ><br>hyperglycemia_threshold]<br><br># Find the time window with the highest average CGM<br>max_avg_cgm_per_day = intraday_hyperglycemia.groupby('Date')['Average_CGM'].idxmax()<br>intraday_trends = intraday_hyperglycemia.loc[max_avg_cgm_per_day]<br><br>intraday_trends.sort_values(by='Average_CGM', ascending=False).head()<br><br>*** [PAUSE] ***<br><br># Filter data for severe hyperglycemia readings<br>severe_hyperglycemia_data = cgm_data[cgm_data['CGM'] > severe_hyperglycemia_threshold]<br><br># Identify consecutive readings spanning at least an hour (12 or more consecutive readings)<br>severe_hyperglycemia_data['ConsecutiveIndex'] = (severe_hyperglycemia_data['DateTime'].diff() !=<br>pd.Timedelta(minutes=5)).cumsum()<br>prolonged_events = severe_hyperglycemia_data.groupby(['Date', 'ConsecutiveIndex']).filter(lambda x: len(x) >=<br>12)<br><br># Extract the start and end times of these events to identify the time of day<br>prolonged_events_summary = prolonged_events.groupby(['Date', 'ConsecutiveIndex']).agg(<br>    Start_Time=('DateTime', 'min'),<br>    End_Time=('DateTime', 'max')<br>).reset_index()<br><br># Map the start times to time windows<br>prolonged_events_summary['TimeOfDay'] =<br>prolonged_events_summary['Start_Time'].dt.hour.apply(map_to_time_window)<br><br>prolonged_events_summary.drop(columns=['ConsecutiveIndex'], inplace=True)<br><br>prolonged_events_summary</pre> |
| | Hypoglycemia | <pre># Define hypoglycemia thresholds<br>hypoglycemia_threshold = 70<br>severe_hypoglycemia_threshold = 54<br><br># Group the data by Date and calculate the percentage of readings below the hypoglycemia thresholds<br>daily_hypoglycemia = cgm_data.groupby('Date').apply(<br>    lambda x: pd.Series({<br>        'Percent_below_70': (x['CGM'] < hypoglycemia_threshold).mean() * 100,<br>        'Percent_below_54': (x['CGM'] < severe_hypoglycemia_threshold).mean() * 100,<br>        'Min_CGM': x['CGM'].min(),<br>    })<br>).reset_index()</pre> |

<table>
<tr><td></td><td></td><td>

```
# Find days with notable hypoglycemia
notable_hypoglycemia_days = daily_hypoglycemia[(daily_hypoglycemia['Percent_below_70'] > 0) |
                                               (daily_hypoglycemia['Percent_below_54'] > 0)]

notable_hypoglycemia_days

*** [PAUSE] ***

# Calculate the average and minimum CGM for each time window across all days for hypoglycemia analysis
intraday_hypoglycemia = cgm_data.groupby(['Date', 'TimeWindow']).agg(
    Average_CGM=('CGM', 'mean'),
    Min_CGM=('CGM', 'min')
).reset_index()

# Identify time windows with hypoglycemia
intraday_hypoglycemia_min = intraday_hypoglycemia[intraday_hypoglycemia['Min_CGM'] < hypoglycemia_threshold]

# Find the time window with the lowest average CGM that still indicates hypoglycemia
min_avg_cgm_per_day = intraday_hypoglycemia.groupby('Date')['Average_CGM'].idxmin()
intraday_hypoglycemia_trends = intraday_hypoglycemia.loc[min_avg_cgm_per_day]

intraday_hypoglycemia_min.sort_values(by='Min_CGM', ascending=True).head()

*** [PAUSE] ***

# Filter data for hypoglycemia readings
hypoglycemia_data = cgm_data[cgm_data['CGM'] < hypoglycemia_threshold]

# Identify consecutive readings indicating prolonged hypoglycemia
hypoglycemia_data['ConsecutiveIndex'] = (hypoglycemia_data['DateTime'].diff() !=
pd.Timedelta(minutes=5)).cumsum()
prolonged_hypo_events = hypoglycemia_data.groupby(['Date', 'ConsecutiveIndex']).filter(lambda x: len(x) >=
12)

# Extract the start and end times of these hypoglycemia events to identify the time of day
prolonged_hypo_summary = prolonged_hypo_events.groupby(['Date', 'ConsecutiveIndex']).agg(
    Start_Time=('DateTime', 'min'),
    End_Time=('DateTime', 'max')
).reset_index()

# Check if any prolonged events were found
prolonged_hypo_summary.drop(columns=['ConsecutiveIndex'], inplace=True)

prolonged_hypo_summary
```

</td></tr>
<tr><td></td><td>Glycemic Variability</td><td>

```
# Calculate the coefficient of variation (CV) for each day
daily_glycemic_variability = cgm_data.groupby('Date').apply(
    lambda x: pd.Series({
        'Mean_CGM': x['CGM'].mean(),
        'StdDev_CGM': x['CGM'].std(),
        'CV': (x['CGM'].std() / x['CGM'].mean()) * 100  # CV formula
    })
).reset_index()

# Identify days with a high coefficient of variation (CV > 36%)
high_cv_days = daily_glycemic_variability[daily_glycemic_variability['CV'] > 36]

high_cv_days

*** [PAUSE] ***

# Calculate the interquartile range (IQR) for each time window across all days
intraday_iqr = cgm_data.groupby('TimeWindow').apply(
    lambda x: x['CGM'].quantile(0.75) - x['CGM'].quantile(0.25)
).reset_index(name='IQR')

intraday_iqr.sort_values(by='IQR', ascending=False)
```

</td></tr>
<tr><td colspan="3"></td></tr>
<tr><td>Case 4</td><td>Data Quality</td><td>

```
# Convert 'Time' column to datetime format to extract unique days and calculate the period
data['DateTime'] = pd.to_datetime(data['Time'])

# Count unique days
unique_days = data['DateTime'].dt.date.nunique()

# Calculate total number of readings
total_readings = len(data)

# Expected number of readings per day is 288 (since readings are every 5 minutes)
expected_readings_per_day = 288
```

</td></tr>
</table>

<table>
<tr><td></td><td></td><td><pre><code># Calculate expected total readings for the observed period
expected_total_readings = expected_readings_per_day * unique_days

# Calculate CGM activity percentage
cgm_activity_percentage = (total_readings / expected_total_readings) * 100

# Assess quality of data
quality_assessment = {
    "unique_days": unique_days,
    "cgm_activity_percentage": cgm_activity_percentage,
    "is_quality_good": unique_days >= 14 and cgm_activity_percentage > 70
}

quality_assessment</code></pre></td></tr>
<tr><td></td><td>Hyperglycemia</td><td><pre><code># Define the hyperglycemia thresholds
hyperglycemia_threshold = 180
severe_hyperglycemia_threshold = 250

# Extract date and hour from the DateTime to facilitate analysis
data['Date'] = data['DateTime'].dt.date
data['Hour'] = data['DateTime'].dt.hour

# Define 4-hour time windows and map them to general times of the day
time_window_mapping = {
    0: "Late night", 4: "Early morning", 8: "Morning",
    12: "Afternoon", 16: "Evening", 20: "Night"
}

# Function to map hour to time window
def map_hour_to_window(hour):
    for window_start in sorted(time_window_mapping.keys()):
        if hour < window_start:
            return time_window_mapping[prev_window_start]
        prev_window_start = window_start
    return time_window_mapping[prev_window_start]

# Apply mapping
data['TimeOfDay'] = data['Hour'].apply(map_hour_to_window)

# a. Hyperglycemia Analysis
# Interday Trends: Days with high levels or high percentage of hyperglycemia
daily_hyperglycemia = data[data['CGM'] > hyperglycemia_threshold].groupby('Date').size()
daily_severe_hyperglycemia = data[data['CGM'] > severe_hyperglycemia_threshold].groupby('Date').size()
total_daily_readings = data.groupby('Date').size()

# Calculate percentage of the day spent in hyperglycemia and severe hyperglycemia
daily_hyperglycemia_percentage = (daily_hyperglycemia / total_daily_readings) * 100
daily_severe_hyperglycemia_percentage = (daily_severe_hyperglycemia / total_daily_readings) * 100

# Identify days with notable hyperglycemia
notable_hyperglycemia_days = daily_hyperglycemia_percentage[daily_hyperglycemia_percentage > 70]
notable_severe_hyperglycemia_days =
daily_severe_hyperglycemia_percentage[daily_severe_hyperglycemia_percentage > 0]

# Intraday Trends: Max glucose in each 4-hour window
intraday_trends = data.groupby(['Date', 'TimeOfDay'])['CGM'].max()

# Prolonged Hyperglycemia: Periods over 250 for an hour or more
prolonged_hyperglycemia = data[data['CGM'] > severe_hyperglycemia_threshold].groupby(['Date',
'TimeOfDay']).filter(lambda x: len(x) >= 12)

# Summary of findings for hyperglycemia analysis
hyperglycemia_analysis_summary = {
    "notable_hyperglycemia_days": notable_hyperglycemia_days.to_dict(),
    "notable_severe_hyperglycemia_days": notable_severe_hyperglycemia_days.to_dict(),
    "prolonged_hyperglycemia_events": prolonged_hyperglycemia.groupby(['Date', 'TimeOfDay']).size().to_dict()
}

hyperglycemia_analysis_summary</code></pre></td></tr>
<tr><td></td><td>Hypoglycemia</td><td><pre><code># Define the hypoglycemia thresholds
hypoglycemia_threshold = 70
severe_hypoglycemia_threshold = 54

# b. Hypoglycemia Analysis
# Interday Trends: Days with low levels or high percentage of hypoglycemia
daily_hypoglycemia = data[data['CGM'] < hypoglycemia_threshold].groupby('Date').size()
daily_severe_hypoglycemia = data[data['CGM'] < severe_hypoglycemia_threshold].groupby('Date').size()

# Calculate percentage of the day spent in hypoglycemia and severe hypoglycemia
daily_hypoglycemia_percentage = (daily_hypoglycemia / total_daily_readings) * 100
daily_severe_hypoglycemia_percentage = (daily_severe_hypoglycemia / total_daily_readings) * 100</code></pre></td></tr>
</table>

| | | |
|---|---|---|
| | | (code below) |

```
# Identify days with notable hypoglycemia
notable_hypoglycemia_days = daily_hypoglycemia_percentage[daily_hypoglycemia_percentage > 0]
notable_severe_hypoglycemia_days = daily_severe_hypoglycemia_percentage[daily_severe_hypoglycemia_percentage
> 0]

# Intraday Trends: Times of the day when hypoglycemia occurs
intraday_hypoglycemia_trends = data[data['CGM'] < hypoglycemia_threshold].groupby(['Date',
'TimeOfDay']).size()

# Prolonged Hypoglycemia: Periods below 70 for more than 60 minutes, or below 54 for more than 15 minutes
prolonged_hypoglycemia_70 = data[data['CGM'] < hypoglycemia_threshold].groupby(['Date',
'TimeOfDay']).filter(lambda x: len(x) > 12)
prolonged_hypoglycemia_54 = data[data['CGM'] < severe_hypoglycemia_threshold].groupby(['Date',
'TimeOfDay']).filter(lambda x: len(x) > 3)

# Summary of findings for hypoglycemia analysis
hypoglycemia_analysis_summary = {
    "notable_hypoglycemia_days": notable_hypoglycemia_days.to_dict(),
    "notable_severe_hypoglycemia_days": notable_severe_hypoglycemia_days.to_dict(),
    "prolonged_hypoglycemia_events_70": prolonged_hypoglycemia_70.groupby(['Date',
'TimeOfDay']).size().to_dict(),
    "prolonged_hypoglycemia_events_54": prolonged_hypoglycemia_54.groupby(['Date',
'TimeOfDay']).size().to_dict()
}

hypoglycemia_analysis_summary

*** [ERROR] ***

# Re-importing pandas after a reset
import pandas as pd

# Re-load the uploaded CSV file
file_path = '/mnt/data/date_correctedmild_adult#006.csv'
data = pd.read_csv(file_path)

# Convert 'Time' column to datetime format to facilitate analysis
data['DateTime'] = pd.to_datetime(data['Time'])

# Define the hypoglycemia thresholds
hypoglycemia_threshold = 70
severe_hypoglycemia_threshold = 54

# Extract date and hour from the DateTime for analysis
data['Date'] = data['DateTime'].dt.date
data['Hour'] = data['DateTime'].dt.hour

# Define 4-hour time windows and map them to general times of the day
time_window_mapping = {
    0: "Late night", 4: "Early morning", 8: "Morning",
    12: "Afternoon", 16: "Evening", 20: "Night"
}

def map_hour_to_window(hour):
    for window_start in sorted(time_window_mapping.keys()):
        if hour < window_start:
            return time_window_mapping[prev_window_start]
        prev_window_start = window_start
    return time_window_mapping[prev_window_start]

data['TimeOfDay'] = data['Hour'].apply(map_hour_to_window)

# Proceed with hypoglycemia analysis

# Interday trends
daily_hypoglycemia_counts = data[data['CGM'] < hypoglycemia_threshold].groupby('Date')['CGM'].count()
daily_severe_hypoglycemia_counts = data[data['CGM'] <
severe_hypoglycemia_threshold].groupby('Date')['CGM'].count()

# Calculate the total number of readings per day for normalization
total_readings_per_day = data.groupby('Date')['CGM'].count()

# Calculate the percentage of the day spent in hypoglycemia and severe hypoglycemia
percentage_daily_hypoglycemia = (daily_hypoglycemia_counts / total_readings_per_day) * 100
percentage_daily_severe_hypoglycemia = (daily_severe_hypoglycemia_counts / total_readings_per_day) * 100

# Intraday trends
intraday_hypoglycemia = data[data['CGM'] < hypoglycemia_threshold].groupby(['TimeOfDay'])['CGM'].count()
intraday_severe_hypoglycemia = data[data['CGM'] <
severe_hypoglycemia_threshold].groupby(['TimeOfDay'])['CGM'].count()

# Prolonged hypoglycemia
prolonged_hypoglycemia = data[data['CGM'] < hypoglycemia_threshold]
prolonged_severe_hypoglycemia = data[data['CGM'] < severe_hypoglycemia_threshold]

# Group by Date and TimeOfDay for aggregation
prolonged_hypoglycemia_groups = prolonged_hypoglycemia.groupby(['Date', 'TimeOfDay'])['CGM'].count()
prolonged_severe_hypoglycemia_groups = prolonged_severe_hypoglycemia.groupby(['Date',
```

```
'TimeOfDay'])['CGM'].count()

# Filter for periods longer than the thresholds
prolonged_hypoglycemia_events = prolonged_hypoglycemia_groups[prolonged_hypoglycemia_groups > 12] # More than 60 minutes
prolonged_severe_hypoglycemia_events =
prolonged_severe_hypoglycemia_groups[prolonged_severe_hypoglycemia_groups > 3] # More than 15 minutes

hypoglycemia_analysis = {
    "percentage_daily_hypoglycemia": percentage_daily_hypoglycemia.to_dict(),
    "percentage_daily_severe_hypoglycemia": percentage_daily_severe_hypoglycemia.to_dict(),
    "prolonged_hypoglycemia_events": prolonged_hypoglycemia_events.to_dict(),
    "prolonged_severe_hypoglycemia_events": prolonged_severe_hypoglycemia_events.to_dict(),
}

hypoglycemia_analysis
```

Glycemic Variability

```
# Calculate the coefficient of variation (CV) for each day
daily_cv = data.groupby('Date')['CGM'].std() / data.groupby('Date')['CGM'].mean() * 100

# Calculate the interquartile range (IQR) for each 24-hour period across the dataset
daily_iqr = data.groupby(['Date'])['CGM'].quantile(0.75) - data.groupby(['Date'])['CGM'].quantile(0.25)

# Calculate the IQR for each time window of the day across the dataset
time_window_iqr = data.groupby(['TimeOfDay'])['CGM'].quantile(0.75) -
data.groupby(['TimeOfDay'])['CGM'].quantile(0.25)

glycemic_variability_analysis = {
    "daily_coefficient_of_variation": daily_cv.to_dict(),
    "daily_interquartile_range": daily_iqr.to_dict(),
    "time_window_interquartile_range": time_window_iqr.to_dict(),
}

glycemic_variability_analysis
```

Case 5

Data Quality

```
from datetime import datetime

# Convert 'day' to datetime for accurate analysis
cgm_data['day'] = pd.to_datetime(cgm_data['day'])

# Count the number of unique days in the dataset
unique_days = cgm_data['day'].nunique()

# Calculate the total possible readings in 14 days (assuming data is recorded every 5 minutes)
total_possible_readings = 14 * 24 * 60 / 5

# Calculate the actual number of readings in the dataset
actual_readings = len(cgm_data)

# Calculate the percentage of CGM activity time
cgm_activity_percentage = (actual_readings / total_possible_readings) * 100

unique_days, cgm_activity_percentage
```

Hyperglycemia

```
import numpy as np

# Define the hyperglycemia thresholds
hyperglycemia_threshold = 180
severe_hyperglycemia_threshold = 250

# Add a column for datetime combining 'day' and 'Time'
cgm_data['datetime'] = pd.to_datetime(cgm_data['day'].astype(str) + ' ' + cgm_data['Time'])

# Function to classify time of day into morning, afternoon, etc.
def classify_time_of_day(hour):
    if 0 <= hour < 6:
        return 'Night'
    elif 6 <= hour < 12:
        return 'Morning'
    elif 12 <= hour < 18:
        return 'Afternoon'
    else:
        return 'Evening'

# Add a column for time of day
cgm_data['time_of_day'] = cgm_data['datetime'].dt.hour.apply(classify_time_of_day)

# Interday Trends: Calculate percentage of day spent in hyperglycemia and severe hyperglycemia
```

| | | |
|---|---|---|
| | | interday_hyperglycemia = cgm_data.groupby('day').apply(lambda x: (x['CGM'] > hyperglycemia_threshold).mean() * 100)<br>interday_severe_hyperglycemia = cgm_data.groupby('day').apply(lambda x: (x['CGM'] > severe_hyperglycemia_threshold).mean() * 100)<br><br># Intraday Trends: Calculate average and maximum glucose for each time window<br>intraday_trends = cgm_data.groupby(['day', 'time_of_day']).agg({'CGM': ['mean', 'max']})<br><br># Prolonged Hyperglycemia: Identify periods where glucose is above 250 for over an hour<br>cgm_data['severe_hyperglycemia'] = cgm_data['CGM'] > severe_hyperglycemia_threshold<br>prolonged_hyperglycemia = cgm_data.groupby(['day', 'time_of_day']).apply(lambda x: (x['severe_hyperglycemia'].rolling(window=12).sum() >= 12).any())<br><br># Prepare data for summary<br>interday_summary = interday_hyperglycemia.describe()<br>intraday_summary = intraday_trends.describe()<br>prolonged_hyperglycemia_summary = prolonged_hyperglycemia.describe()<br><br>interday_summary, intraday_summary, prolonged_hyperglycemia_summary<br><br>*** [ERROR] ***<br><br># There seems to be an issue with the datetime conversion<br># Let's correct this by ensuring that the 'datetime' column is properly formatted<br><br># Re-examine the 'Time' and 'day' columns to identify the correct format<br>cgm_data[['day', 'Time']].head()<br><br>*** [PAUSE] ***<br><br># Correct the 'datetime' column by using only the 'Time' column<br>cgm_data['datetime'] = pd.to_datetime(cgm_data['Time'])<br><br># Add a column for time of day<br>cgm_data['time_of_day'] = cgm_data['datetime'].dt.hour.apply(classify_time_of_day)<br><br># Re-attempt the analysis with corrected datetime<br><br># Interday Trends: Calculate percentage of day spent in hyperglycemia and severe hyperglycemia<br>interday_hyperglycemia = cgm_data.groupby(cgm_data['datetime'].dt.date).apply(lambda x: (x['CGM'] > hyperglycemia_threshold).mean() * 100)<br>interday_severe_hyperglycemia = cgm_data.groupby(cgm_data['datetime'].dt.date).apply(lambda x: (x['CGM'] > severe_hyperglycemia_threshold).mean() * 100)<br><br># Intraday Trends: Calculate average and maximum glucose for each time window<br>intraday_trends = cgm_data.groupby(['datetime', 'time_of_day']).agg({'CGM': ['mean', 'max']})<br><br># Prolonged Hyperglycemia: Identify periods where glucose is above 250 for over an hour<br>prolonged_hyperglycemia = cgm_data.groupby(['datetime', 'time_of_day']).apply(lambda x: (x['severe_hyperglycemia'].rolling(window=12).sum() >= 12).any())<br><br># Prepare data for summary<br>interday_summary = interday_hyperglycemia.describe()<br>intraday_summary = intraday_trends.describe()<br>prolonged_hyperglycemia_summary = prolonged_hyperglycemia.describe()<br><br>interday_summary, intraday_summary, prolonged_hyperglycemia_summary<br><br>*** [ERROR] ***<br><br># Correcting the issue by creating the 'severe_hyperglycemia' column<br>cgm_data['severe_hyperglycemia'] = cgm_data['CGM'] > severe_hyperglycemia_threshold<br><br># Re-attempt the analysis with the corrected data<br><br># Interday Trends: Calculate percentage of day spent in hyperglycemia and severe hyperglycemia<br>interday_hyperglycemia = cgm_data.groupby(cgm_data['datetime'].dt.date).apply(lambda x: (x['CGM'] > hyperglycemia_threshold).mean() * 100)<br>interday_severe_hyperglycemia = cgm_data.groupby(cgm_data['datetime'].dt.date).apply(lambda x: (x['CGM'] > severe_hyperglycemia_threshold).mean() * 100)<br><br># Intraday Trends: Calculate average and maximum glucose for each time window<br>intraday_trends = cgm_data.groupby(['datetime', 'time_of_day']).agg({'CGM': ['mean', 'max']})<br><br># Prolonged Hyperglycemia: Identify periods where glucose is above 250 for over an hour<br>prolonged_hyperglycemia = cgm_data.groupby(['datetime', 'time_of_day']).apply(lambda x: (x['severe_hyperglycemia'].rolling(window=12).sum() >= 12).any())<br><br># Prepare data for summary<br>interday_summary = interday_hyperglycemia.describe()<br>intraday_summary = intraday_trends.describe()<br>prolonged_hyperglycemia_summary = prolonged_hyperglycemia.describe()<br><br>interday_summary, intraday_summary, prolonged_hyperglycemia_summary |

| | | |
|---|---|---|
| | Hypoglycemia | <pre># Define the hypoglycemia thresholds<br>mild_hypoglycemia_threshold = 70<br>severe_hypoglycemia_threshold = 54<br><br># Interday Trends: Calculate percentage of day spent in hypoglycemia (below 70 and 54 mg/dL)<br>interday_mild_hypoglycemia = cgm_data.groupby(cgm_data['datetime'].dt.date).apply(lambda x: (x['CGM'] <<br>mild_hypoglycemia_threshold).mean() * 100)<br>interday_severe_hypoglycemia = cgm_data.groupby(cgm_data['datetime'].dt.date).apply(lambda x: (x['CGM'] <<br>severe_hypoglycemia_threshold).mean() * 100)<br><br># Intraday Trends: Identify if hypoglycemia tends to occur at specific times of the day<br>intraday_hypoglycemia = cgm_data.groupby(['datetime', 'time_of_day']).apply(lambda x: (x['CGM'] <<br>mild_hypoglycemia_threshold).any())<br><br># Prolonged Hypoglycemia: Identify periods where glucose is below 70 for more than 60 minutes, or below 54<br>for more than 15 minutes<br>prolonged_mild_hypoglycemia = cgm_data.groupby(['datetime', 'time_of_day']).apply(lambda x: (x['CGM'] <<br>mild_hypoglycemia_threshold).rolling(window=12).sum() >= 12).any()<br>prolonged_severe_hypoglycemia = cgm_data.groupby(['datetime', 'time_of_day']).apply(lambda x: (x['CGM'] <<br>severe_hypoglycemia_threshold).rolling(window=3).sum() >= 3).any()<br><br># Prepare data for summary<br>interday_mild_hypoglycemia_summary = interday_mild_hypoglycemia.describe()<br>interday_severe_hypoglycemia_summary = interday_severe_hypoglycemia.describe()<br>intraday_hypoglycemia_summary = intraday_hypoglycemia.describe()<br>prolonged_mild_hypoglycemia_summary = prolonged_mild_hypoglycemia.describe()<br>prolonged_severe_hypoglycemia_summary = prolonged_severe_hypoglycemia.describe()<br><br>(interday_mild_hypoglycemia_summary, interday_severe_hypoglycemia_summary, intraday_hypoglycemia_summary,<br>prolonged_mild_hypoglycemia_summary, prolonged_severe_hypoglycemia_summary)<br><br><br>*** [ERROR] ***<br><br># Correcting the analysis for prolonged hypoglycemia<br><br># Prolonged Hypoglycemia: Identify periods where glucose is below 70 for more than 60 minutes, or below 54<br>for more than 15 minutes<br>prolonged_mild_hypoglycemia = cgm_data.groupby(cgm_data['datetime'].dt.date).apply(lambda x: (x['CGM'] <<br>mild_hypoglycemia_threshold).rolling(window=12).sum() >= 12).any()<br>prolonged_severe_hypoglycemia = cgm_data.groupby(cgm_data['datetime'].dt.date).apply(lambda x: (x['CGM'] <<br>severe_hypoglycemia_threshold).rolling(window=3).sum() >= 3).any()<br><br># Prepare data for summary<br>prolonged_mild_hypoglycemia_summary = prolonged_mild_hypoglycemia.describe()<br>prolonged_severe_hypoglycemia_summary = prolonged_severe_hypoglycemia.describe()<br><br>(prolonged_mild_hypoglycemia_summary, prolonged_severe_hypoglycemia_summary)<br><br>*** [ERROR] ***<br><br># Refining the analysis for prolonged hypoglycemia<br><br># Identify specific days and times when glucose is below 70 for more than 60 minutes<br>prolonged_mild_hypoglycemia_events = cgm_data[cgm_data['CGM'] <<br>mild_hypoglycemia_threshold].groupby(cgm_data['datetime'].dt.date).rolling(window=12).sum()<br>prolonged_mild_hypoglycemia_days =<br>prolonged_mild_hypoglycemia_events[prolonged_mild_hypoglycemia_events['CGM'] >=<br>12].index.get_level_values(0).unique()<br><br># Identify specific days and times when glucose is below 54 for more than 15 minutes<br>prolonged_severe_hypoglycemia_events = cgm_data[cgm_data['CGM'] <<br>severe_hypoglycemia_threshold].groupby(cgm_data['datetime'].dt.date).rolling(window=3).sum()<br>prolonged_severe_hypoglycemia_days =<br>prolonged_severe_hypoglycemia_events[prolonged_severe_hypoglycemia_events['CGM'] >=<br>3].index.get_level_values(0).unique()<br><br>prolonged_mild_hypoglycemia_days, prolonged_severe_hypoglycemia_days</pre> |
| | Glycemic Variability | <pre># Calculate Coefficient of Variation (CV) for each day<br>daily_cv = cgm_data.groupby(cgm_data['datetime'].dt.date).apply(lambda x: (x['CGM'].std() / x['CGM'].mean())<br>* 100)<br><br># Identify days with high coefficient of variation<br>high_cv_days = daily_cv[daily_cv > 36]<br><br># Calculate the Interquartile Range (IQR) for each time window of the day<br>daily_iqr = cgm_data.groupby(['datetime', 'time_of_day'])['CGM'].agg(lambda x: x.quantile(0.75) -<br>x.quantile(0.25))<br><br># Identify times of the day with higher IQR<br>high_iqr_times = daily_iqr.groupby('time_of_day').mean()<br><br>high_cv_days, high_iqr_times</pre> |

| | | |
|---|---|---|
| | | |

**Case 6** — **Data Quality**

```
from datetime import datetime

# Converting the 'Time' column to datetime format
cgm_data['Time'] = pd.to_datetime(cgm_data['Time'], format='%m/%d/%y %H:%M')

# Checking the total number of unique days in the dataset
unique_days = cgm_data['Time'].dt.date.nunique()

# Calculating the total number of readings and the expected number of readings for 14 days
# Assuming CGM data is collected every 5 minutes, there should be 288 readings per day (24*60/5)
total_readings = len(cgm_data)
expected_readings_per_day = 24 * 60 / 5
expected_readings_14_days = expected_readings_per_day * 14

# Calculating the percentage of CGM activity time
activity_percentage = (total_readings / expected_readings_14_days) * 100

unique_days, activity_percentage
```

**Hyperglycemia**

```
import numpy as np

# Defining the hyperglycemia thresholds
hyperglycemia_threshold = 180
severe_hyperglycemia_threshold = 250

# Adding a column for the date and the hour to facilitate analysis
cgm_data['Date'] = cgm_data['Time'].dt.date
cgm_data['Hour'] = cgm_data['Time'].dt.hour

# Breaking the day into 4-hour time windows and mapping them to general times of the day
def map_time_window(hour):
    if 0 <= hour < 4:
        return "Late night"
    elif 4 <= hour < 8:
        return "Early morning"
    elif 8 <= hour < 12:
        return "Morning"
    elif 12 <= hour < 16:
        return "Afternoon"
    elif 16 <= hour < 20:
        return "Evening"
    else:
        return "Night"

cgm_data['Time Window'] = cgm_data['Hour'].apply(map_time_window)

# Analyzing for hyperglycemia
# 1. Interday Trends
interday_hyperglycemia = cgm_data[cgm_data['CGM'] >
hyperglycemia_threshold].groupby('Date')['CGM'].agg(['count', 'mean'])
interday_hyperglycemia['Percentage of Day'] = (interday_hyperglycemia['count'] / expected_readings_per_day) *
100
interday_severe_hyperglycemia = cgm_data[cgm_data['CGM'] >
severe_hyperglycemia_threshold].groupby('Date')['CGM'].agg(['count', 'mean'])
interday_severe_hyperglycemia['Percentage of Day'] = (interday_severe_hyperglycemia['count'] /
expected_readings_per_day) * 100

# 2. Intraday Trends
intraday_hyperglycemia = cgm_data.groupby('Time Window')['CGM'].agg(['max', 'mean'])

# 3. Prolonged Hyperglycemia
# Checking for periods where glucose readings are over 250 for an hour or more
cgm_data['Severe Hyperglycemia'] = cgm_data['CGM'] > severe_hyperglycemia_threshold
prolonged_hyperglycemia = cgm_data.groupby(['Date', 'Time Window'])['Severe
Hyperglycemia'].rolling(window=12).sum()
prolonged_hyperglycemia = prolonged_hyperglycemia.reset_index()
prolonged_hyperglycemia = prolonged_hyperglycemia[prolonged_hyperglycemia['Severe Hyperglycemia'] >= 12]

# Selecting relevant data to summarize
interday_hyperglycemia_summary = interday_hyperglycemia.sort_values(by='Percentage of Day',
ascending=False).head()
interday_severe_hyperglycemia_summary = interday_severe_hyperglycemia.sort_values(by='Percentage of Day',
ascending=False).head()
intraday_hyperglycemia_summary = intraday_hyperglycemia.sort_values(by='max', ascending=False).head()
prolonged_hyperglycemia_summary = prolonged_hyperglycemia.drop_duplicates(subset=['Date', 'Time Window'])

interday_hyperglycemia_summary, interday_severe_hyperglycemia_summary, intraday_hyperglycemia_summary,
prolonged_hyperglycemia_summary.head()
```

**Hypoglycemia**

```
# Defining the hypoglycemia thresholds
```

| | | |
|---|---|---|
| | | ```<br>hypoglycemia_threshold = 70<br>severe_hypoglycemia_threshold = 54<br>``` |

```
hypoglycemia_threshold = 70
severe_hypoglycemia_threshold = 54

# Analyzing for hypoglycemia
# 1. Interday Trends
interday_hypoglycemia = cgm_data[cgm_data['CGM'] <
hypoglycemia_threshold].groupby('Date')['CGM'].agg(['count', 'mean'])
interday_hypoglycemia['Percentage of Day'] = (interday_hypoglycemia['count'] / expected_readings_per_day) *
100
interday_severe_hypoglycemia = cgm_data[cgm_data['CGM'] <
severe_hypoglycemia_threshold].groupby('Date')['CGM'].agg(['count', 'mean'])
interday_severe_hypoglycemia['Percentage of Day'] = (interday_severe_hypoglycemia['count'] /
expected_readings_per_day) * 100

# 2. Intraday Trends
intraday_hypoglycemia = cgm_data.groupby('Time Window')['CGM'].agg(['min', 'mean'])

# 3. Prolonged Hypoglycemia
# Checking for periods where glucose readings are below 70 for more than 60 minutes, and below 54 for more
than 15 minutes
cgm_data['Hypoglycemia'] = cgm_data['CGM'] < hypoglycemia_threshold
cgm_data['Severe Hypoglycemia'] = cgm_data['CGM'] < severe_hypoglycemia_threshold
prolonged_hypoglycemia = cgm_data.groupby(['Date', 'Time Window'])['Hypoglycemia'].rolling(window=12).sum()
prolonged_hypoglycemia = prolonged_hypoglycemia.reset_index()
prolonged_hypoglycemia = prolonged_hypoglycemia[prolonged_hypoglycemia['Hypoglycemia'] >= 12]

prolonged_severe_hypoglycemia = cgm_data.groupby(['Date', 'Time Window'])['Severe
Hypoglycemia'].rolling(window=3).sum()
prolonged_severe_hypoglycemia = prolonged_severe_hypoglycemia.reset_index()
prolonged_severe_hypoglycemia = prolonged_severe_hypoglycemia[prolonged_severe_hypoglycemia['Severe
Hypoglycemia'] >= 3]

# Selecting relevant data to summarize
interday_hypoglycemia_summary = interday_hypoglycemia.sort_values(by='Percentage of Day',
ascending=False).head()
interday_severe_hypoglycemia_summary = interday_severe_hypoglycemia.sort_values(by='Percentage of Day',
ascending=False).head()
intraday_hypoglycemia_summary = intraday_hypoglycemia.sort_values(by='min').head()
prolonged_hypoglycemia_summary = prolonged_hypoglycemia.drop_duplicates(subset=['Date', 'Time Window'])
prolonged_severe_hypoglycemia_summary = prolonged_severe_hypoglycemia.drop_duplicates(subset=['Date', 'Time
Window'])

interday_hypoglycemia_summary, interday_severe_hypoglycemia_summary, intraday_hypoglycemia_summary,
prolonged_hypoglycemia_summary.head(), prolonged_severe_hypoglycemia_summary.head()

*** [ERROR] ***

# Re-importing pandas and reloading the dataset due to execution state reset
import pandas as pd

# Reloading the dataset
file_path = '/mnt/data/date_correcteddinner_adult#006.csv'
cgm_data = pd.read_csv(file_path)

# Converting the 'Time' column to datetime format
cgm_data['Time'] = pd.to_datetime(cgm_data['Time'], format='%m/%d/%y %H:%M')

# Adding necessary columns for analysis
cgm_data['Date'] = cgm_data['Time'].dt.date
cgm_data['Hour'] = cgm_data['Time'].dt.hour
cgm_data['Time Window'] = cgm_data['Hour'].apply(lambda hour:
    "Late night" if 0 <= hour < 4 else
    "Early morning" if 4 <= hour < 8 else
    "Morning" if 8 <= hour < 12 else
    "Afternoon" if 12 <= hour < 16 else
    "Evening" if 16 <= hour < 20 else
    "Night")

# Defining hypoglycemia thresholds
hypoglycemia_threshold = 70
severe_hypoglycemia_threshold = 54

# Analyzing for hypoglycemia
# Interday and Intraday Trends
interday_hypoglycemia = cgm_data[cgm_data['CGM'] <
hypoglycemia_threshold].groupby('Date')['CGM'].agg(['count', 'mean'])
interday_hypoglycemia['Percentage of Day'] = (interday_hypoglycemia['count'] / (24 * 60 / 5)) * 100
interday_severe_hypoglycemia = cgm_data[cgm_data['CGM'] <
severe_hypoglycemia_threshold].groupby('Date')['CGM'].agg(['count', 'mean'])
interday_severe_hypoglycemia['Percentage of Day'] = (interday_severe_hypoglycemia['count'] / (24 * 60 / 5)) *
100
intraday_hypoglycemia = cgm_data.groupby('Time Window')['CGM'].agg(['min', 'mean'])

# Prolonged Hypoglycemia
cgm_data['Hypoglycemia'] = cgm_data['CGM'] < hypoglycemia_threshold
cgm_data['Severe Hypoglycemia'] = cgm_data['CGM'] < severe_hypoglycemia_threshold
prolonged_hypoglycemia = cgm_data.groupby(['Date', 'Time
Window'])['Hypoglycemia'].rolling(window=12).sum().reset_index(name='Count')
prolonged_hypoglycemia = prolonged_hypoglycemia[prolonged_hypoglycemia['Count'] >= 12]
```

```
prolonged_severe_hypoglycemia = cgm_data.groupby(['Date', 'Time Window'])['Severe
Hypoglycemia'].rolling(window=3).sum().reset_index(name='Count')
prolonged_severe_hypoglycemia = prolonged_severe_hypoglycemia[prolonged_severe_hypoglycemia['Count'] >= 3]

# Selecting relevant data to summarize
interday_hypoglycemia_summary = interday_hypoglycemia.sort_values(by='Percentage of Day',
ascending=False).head()
interday_severe_hypoglycemia_summary = interday_severe_hypoglycemia.sort_values(by='Percentage of Day',
ascending=False).head()
intraday_hypoglycemia_summary = intraday_hypoglycemia.sort_values(by='min').head()
prolonged_hypoglycemia_summary = prolonged_hypoglycemia.drop_duplicates(subset=['Date', 'Time Window'])
prolonged_severe_hypoglycemia_summary = prolonged_severe_hypoglycemia.drop_duplicates(subset=['Date', 'Time
Window'])

interday_hypoglycemia_summary, interday_severe_hypoglycemia_summary, intraday_hypoglycemia_summary,
prolonged_hypoglycemia_summary.head(), prolonged_severe_hypoglycemia_summary.head()
```

Glycemic Variability

```
# Re-importing numpy and calculating the coefficient of variation and interquartile range
import numpy as np

# Calculating Coefficient of Variation (CV) for each day
daily_cv = cgm_data.groupby('Date')['CGM'].agg(['mean', 'std'])
daily_cv['Coefficient of Variation'] = (daily_cv['std'] / daily_cv['mean']) * 100

# Calculating Interquartile Range (IQR) for each 24-hour time window
iqr_by_time_window = cgm_data.groupby('Time Window')['CGM'].agg(lambda x: np.percentile(x, 75) -
np.percentile(x, 25))

# Selecting relevant data to summarize
high_cv_days = daily_cv[daily_cv['Coefficient of Variation'] > 36]
high_cv_days_summary = high_cv_days.sort_values(by='Coefficient of Variation', ascending=False)
iqr_by_time_window_summary = iqr_by_time_window.sort_values(ascending=False)

high_cv_days_summary, iqr_by_time_window_summary
```

Case 7

Data Quality

```
from datetime import datetime

# Convert the 'Time' column to datetime format
cgm_data['Time'] = pd.to_datetime(cgm_data['Time'], format='%m/%d/%y %H:%M')

# Check the unique days in the dataset
unique_days = cgm_data['Time'].dt.date.nunique()

# Calculate the total possible readings (assuming 5-minute intervals in 24 hours)
total_readings_per_day = 24 * 60 / 5  # 288 readings per day
total_possible_readings = total_readings_per_day * unique_days

# Calculate the actual number of readings
actual_readings = len(cgm_data)

# Calculate the percentage of CGM activity time
percentage_activity_time = (actual_readings / total_possible_readings) * 100

unique_days, percentage_activity_time
```

Hyperglycemia

```
import numpy as np

# Define the hyperglycemia thresholds
hyperglycemia_threshold = 180
severe_hyperglycemia_threshold = 250

# Adding a column for date and a column for the 4-hour time window
cgm_data['Date'] = cgm_data['Time'].dt.date
cgm_data['TimeWindow'] = cgm_data['Time'].dt.hour // 4

# Map the time window to general times of the day
time_window_map = {
    0: "Late Night",
    1: "Early Morning",
    2: "Morning",
    3: "Afternoon",
    4: "Evening",
    5: "Night"
}
cgm_data['TimeOfDay'] = cgm_data['TimeWindow'].map(time_window_map)

# Interday Trends
# Group by date and calculate the percentage of readings above each threshold
```

<table>
<tr>
<td></td>
<td></td>
<td><pre>daily_hyperglycemia = cgm_data.groupby('Date').apply(
    lambda x: pd.Series({
        'PercentAbove180': np.mean(x['CGM'] > hyperglycemia_threshold) * 100,
        'PercentAbove250': np.mean(x['CGM'] > severe_hyperglycemia_threshold) * 100
    })
)

# Intraday Trends
# Group by time of day and calculate average glucose level
intraday_avg_glucose = cgm_data.groupby('TimeOfDay')['CGM'].mean()

# Prolonged Hyperglycemia
# Check for periods where glucose readings are over 250 for an hour or more
prolonged_hyperglycemia = cgm_data[cgm_data['CGM'] > severe_hyperglycemia_threshold].groupby(['Date',
'TimeOfDay']).size()
prolonged_hyperglycemia = prolonged_hyperglycemia[prolonged_hyperglycemia >= 12]  # 12 consecutive readings

daily_hyperglycemia, intraday_avg_glucose, prolonged_hyperglycemia</pre></td>
</tr>
<tr>
<td></td>
<td>Hypoglycemia</td>
<td><pre># Define the hypoglycemia thresholds
hypoglycemia_threshold = 70
severe_hypoglycemia_threshold = 54

# Interday Trends
# Group by date and calculate the percentage of readings below each threshold
daily_hypoglycemia = cgm_data.groupby('Date').apply(
    lambda x: pd.Series({
        'PercentBelow70': np.mean(x['CGM'] < hypoglycemia_threshold) * 100,
        'PercentBelow54': np.mean(x['CGM'] < severe_hypoglycemia_threshold) * 100
    })
)

# Intraday Trends
# Group by time of day and check for hypoglycemia
intraday_hypoglycemia = cgm_data.groupby('TimeOfDay').apply(
    lambda x: pd.Series({
        'PercentBelow70': np.mean(x['CGM'] < hypoglycemia_threshold) * 100,
        'PercentBelow54': np.mean(x['CGM'] < severe_hypoglycemia_threshold) * 100
    })
)

# Prolonged Hypoglycemia
# Check for periods where glucose readings are below 70 for more than 60 minutes or below 54 for more than 15
minutes
prolonged_hypoglycemia_70 = cgm_data[cgm_data['CGM'] < hypoglycemia_threshold].groupby(['Date',
'TimeOfDay']).size()
prolonged_hypoglycemia_70 = prolonged_hypoglycemia_70[prolonged_hypoglycemia_70 >= 12]  # 60 minutes

prolonged_hypoglycemia_54 = cgm_data[cgm_data['CGM'] < severe_hypoglycemia_threshold].groupby(['Date',
'TimeOfDay']).size()
prolonged_hypoglycemia_54 = prolonged_hypoglycemia_54[prolonged_hypoglycemia_54 >= 3]  # 15 minutes

daily_hypoglycemia, intraday_hypoglycemia, prolonged_hypoglycemia_70, prolonged_hypoglycemia_54</pre></td>
</tr>
<tr>
<td></td>
<td>Glycemic Variability</td>
<td><pre># Calculate Coefficient of Variation for each day
daily_cv = cgm_data.groupby('Date')['CGM'].std() / cgm_data.groupby('Date')['CGM'].mean() * 100

# Calculate Interquartile Range for each 24-hour period
daily_iqr = cgm_data.groupby(['Date', 'TimeOfDay'])['CGM'].quantile(0.75) - cgm_data.groupby(['Date',
'TimeOfDay'])['CGM'].quantile(0.25)

daily_cv, daily_iqr.groupby('TimeOfDay').mean()</pre></td>
</tr>
<tr>
<td colspan="3"></td>
</tr>
<tr>
<td>Case 8</td>
<td>Data Quality</td>
<td><pre>from datetime import datetime

# Convert 'Time' column to datetime format
data['datetime'] = pd.to_datetime(data['Time'], format='%m/%d/%y %H:%M')

# Calculate the number of unique days in the data
unique_days = data['datetime'].dt.date.nunique()

# Calculate total number of readings and expected readings
total_readings = data.shape[0]
expected_readings_per_day = 24 * 60 / 5  # 5-minute intervals in a day
expected_total_readings = expected_readings_per_day * unique_days

# Calculate CGM activity time as a percentage
cgm_activity_percentage = (total_readings / expected_total_readings) * 100</pre></td>
</tr>
</table>

<table>
<tr><td></td><td></td><td><pre>unique_days, cgm_activity_percentage</pre></td></tr>
<tr><td></td><td>Hyperglycemia</td><td><pre>
# Define hyperglycemia thresholds
hyperglycemia_threshold = 180
severe_hyperglycemia_threshold = 250

# Add a column for the 4-hour time window
data['time_window'] = data['datetime'].dt.hour // 4

# Map the 4-hour time window to general times of the day
time_of_day_mapping = {
    0: "Late night",
    1: "Early morning",
    2: "Morning",
    3: "Afternoon",
    4: "Evening",
    5: "Night"
}
data['time_of_day'] = data['time_window'].map(time_of_day_mapping)

# Analyze hyperglycemia
# 1. Interday trends
daily_hyperglycemia = data[data['CGM'] >
hyperglycemia_threshold].groupby(data['datetime'].dt.date).agg(['count', 'mean', 'max'])
daily_severe_hyperglycemia = data[data['CGM'] >
severe_hyperglycemia_threshold].groupby(data['datetime'].dt.date).agg(['count', 'mean', 'max'])

# 2. Intraday trends
intraday_hyperglycemia = data.groupby('time_of_day')['CGM'].agg(['mean', 'max'])

# 3. Prolonged hyperglycemia
# Identify continuous sequences of severe hyperglycemia readings lasting an hour or more
data['severe_hyperglycemia'] = data['CGM'] > severe_hyperglycemia_threshold
data['group'] = (data['severe_hyperglycemia'] != data['severe_hyperglycemia'].shift()).cumsum()
prolonged_hyperglycemia_groups = data[data['severe_hyperglycemia']].groupby('group').agg(count=('CGM',
'count'), start=('datetime', 'min'), end=('datetime', 'max'))
prolonged_hyperglycemia_events = prolonged_hyperglycemia_groups[prolonged_hyperglycemia_groups['count'] >=
12]

# Since we're not displaying tables as per instructions, let's summarize the findings instead
interday_hyperglycemia_summary = daily_hyperglycemia.shape[0] > 0
interday_severe_hyperglycemia_summary = daily_severe_hyperglycemia.shape[0] > 0
prolonged_hyperglycemia_summary = prolonged_hyperglycemia_events.shape[0] > 0

interday_hyperglycemia_summary, interday_severe_hyperglycemia_summary, prolonged_hyperglycemia_summary
</pre></td></tr>
<tr><td></td><td>Hypoglycemia</td><td><pre>
# Define hypoglycemia thresholds
hypoglycemia_threshold = 70
severe_hypoglycemia_threshold = 54

# Analyze hypoglycemia
# 1. Interday trends
daily_hypoglycemia = data[data['CGM'] <
hypoglycemia_threshold].groupby(data['datetime'].dt.date).agg(['count', 'mean', 'min'])
daily_severe_hypoglycemia = data[data['CGM'] <
severe_hypoglycemia_threshold].groupby(data['datetime'].dt.date).agg(['count', 'mean', 'min'])

# 2. Intraday trends
intraday_hypoglycemia = data.groupby('time_of_day')['CGM'].agg(['mean', 'min'])

# 3. Prolonged hypoglycemia
# Below 70 for more than 60 minutes
data['hypoglycemia'] = data['CGM'] < hypoglycemia_threshold
data['severe_hypoglycemia'] = data['CGM'] < severe_hypoglycemia_threshold
data['hypoglycemia_group'] = (data['hypoglycemia'] != data['hypoglycemia'].shift()).cumsum()
data['severe_hypoglycemia_group'] = (data['severe_hypoglycemia'] !=
data['severe_hypoglycemia'].shift()).cumsum()

prolonged_hypoglycemia_groups = data[data['hypoglycemia']].groupby('hypoglycemia_group').agg(count=('CGM',
'count'), start=('datetime', 'min'), end=('datetime', 'max'))
prolonged_hypoglycemia_events = prolonged_hypoglycemia_groups[prolonged_hypoglycemia_groups['count'] > 12]  #
More than 60 minutes

prolonged_severe_hypoglycemia_groups =
data[data['severe_hypoglycemia']].groupby('severe_hypoglycemia_group').agg(count=('CGM', 'count'),
start=('datetime', 'min'), end=('datetime', 'max'))
prolonged_severe_hypoglycemia_events =
prolonged_severe_hypoglycemia_groups[prolonged_severe_hypoglycemia_groups['count'] > 3]  # More than 15
minutes

# Summarize findings
prolonged_hypoglycemia_summary = prolonged_hypoglycemia_events.shape[0] > 0
prolonged_severe_hypoglycemia_summary = prolonged_severe_hypoglycemia_events.shape[0] > 0
</pre></td></tr>
</table>

```
prolonged_hypoglycemia_summary, prolonged_severe_hypoglycemia_summary
```

**Glycemic Variability**

```
# Calculate coefficient of variation for each day
daily_cgm = data.groupby(data['datetime'].dt.date)['CGM']
daily_coefficient_of_variation = daily_cgm.std() / daily_cgm.mean() * 100

# Calculate interquartile range (IQR) for each 24-hour period
daily_iqr = daily_cgm.quantile(0.75) - daily_cgm.quantile(0.25)

# Calculate IQR for each time of day across all days
intraday_iqr = data.groupby('time_of_day')['CGM'].agg(lambda x: x.quantile(0.75) - x.quantile(0.25))

# Identify days with high coefficient of variation (> 36%)
high_cv_days = daily_coefficient_of_variation[daily_coefficient_of_variation > 36]

# Summarize findings
high_cv_summary = high_cv_days.shape[0] > 0

high_cv_summary, intraday_iqr.describe()


*** [ERROR] ***

# Re-import pandas and reload the data due to code execution reset
import pandas as pd

# Load the data again
file_path = '/mnt/data/date_correctedhealthy_adult#008.csv'
data = pd.read_csv(file_path)

# Convert 'Time' column to datetime format and prepare data for analysis
data['datetime'] = pd.to_datetime(data['Time'], format='%m/%d/%y %H:%M')
data['time_window'] = data['datetime'].dt.hour // 4

# Map the 4-hour time window to general times of the day
time_of_day_mapping = {
    0: "Late night",
    1: "Early morning",
    2: "Morning",
    3: "Afternoon",
    4: "Evening",
    5: "Night"
}
data['time_of_day'] = data['time_window'].map(time_of_day_mapping)

# Calculate coefficient of variation for each day
daily_cgm = data.groupby(data['datetime'].dt.date)['CGM']
daily_coefficient_of_variation = daily_cgm.std() / daily_cgm.mean() * 100

# Calculate interquartile range (IQR) for each 24-hour period
daily_iqr = daily_cgm.quantile(0.75) - daily_cgm.quantile(0.25)

# Calculate IQR for each time of day across all days
intraday_iqr = data.groupby('time_of_day')['CGM'].agg(lambda x: x.quantile(0.75) - x.quantile(0.25))

# Identify days with high coefficient of variation (> 36%)
high_cv_days = daily_coefficient_of_variation[daily_coefficient_of_variation > 36]

# Summarize findings
high_cv_summary = high_cv_days.shape[0] > 0

high_cv_summary, intraday_iqr.describe()
```

**Case 9** — **Data Quality**

```
# Convert 'Time' column to datetime format and extract date
data['DateTime'] = pd.to_datetime(data['Time'])
data['Date'] = data['DateTime'].dt.date

# Count unique days
unique_days = data['Date'].nunique()

# Calculate CGM activity time
# Assuming each record is spaced 5 minutes apart, we can calculate total minutes and then convert to
percentage of the period
total_records = data.shape[0]
total_minutes = total_records * 5
total_days_covered = total_minutes / (24 * 60)
percentage_cgm_activity_time = (total_days_covered / unique_days) * 100 if unique_days else 0

unique_days, percentage_cgm_activity_time
```

Hyperglycemia

```
# Define 4-hour time windows and label them
def assign_time_window(hour):
    if 0 <= hour < 4:
        return "Late night"
    elif 4 <= hour < 8:
        return "Early morning"
    elif 8 <= hour < 12:
        return "Morning"
    elif 12 <= hour < 16:
        return "Afternoon"
    elif 16 <= hour < 20:
        return "Evening"
    else:  # 20 <= hour < 24
        return "Night"

# Extract hour from DateTime and assign time window
data['Hour'] = data['DateTime'].dt.hour
data['TimeWindow'] = data['Hour'].apply(assign_time_window)

# Analyze interday trends for hyperglycemia
# Calculate the percentage of readings above 180 and 250 mg/dL per day
hyperglycemia_threshold = 180
severe_hyperglycemia_threshold = 250

# Calculate daily averages and percentages above thresholds
daily_analysis = data.groupby('Date').agg(
    AvgGlucose=('CGM', 'mean'),
    MaxGlucose=('CGM', 'max'),
    PercentAbove180=('CGM', lambda x: (x > hyperglycemia_threshold).mean() * 100),
    PercentAbove250=('CGM', lambda x: (x > severe_hyperglycemia_threshold).mean() * 100)
).reset_index()

# Identify days with prolonged hyperglycemia (more than 12 consecutive readings above 250)
data['SevereHyperFlag'] = data['CGM'] > severe_hyperglycemia_threshold
prolonged_hyper_events = data[data['SevereHyperFlag']].groupby(['Date', 'TimeWindow']).filter(lambda x:
len(x) >= 12)

# Extract information about prolonged hyperglycemia events
prolonged_hyper_days_times = prolonged_hyper_events.groupby(['Date',
'TimeWindow']).size().reset_index(name='Count')

daily_analysis, prolonged_hyper_days_times
```

Hypoglycemia

```
# Define hypoglycemia thresholds
hypoglycemia_threshold = 70
severe_hypoglycemia_threshold = 54

# Calculate daily percentages below thresholds and identify prolonged hypoglycemia events
daily_hypoglycemia_analysis = data.groupby('Date').agg(
    AvgGlucose=('CGM', 'mean'),
    MinGlucose=('CGM', 'min'),
    PercentBelow70=('CGM', lambda x: (x < hypoglycemia_threshold).mean() * 100),
    PercentBelow54=('CGM', lambda x: (x < severe_hypoglycemia_threshold).mean() * 100)
).reset_index()

# Identify periods of prolonged hypoglycemia (below 70 for more than 60 minutes, below 54 for more than 15
minutes)
data['HypoFlag70'] = data['CGM'] < hypoglycemia_threshold
data['HypoFlag54'] = data['CGM'] < severe_hypoglycemia_threshold

# For prolonged hypoglycemia, identify consecutive readings fulfilling the criteria
prolonged_hypo_70 = data[data['HypoFlag70']].groupby(['Date', 'TimeWindow']).filter(lambda x: len(x) > 12) #
More than 60 minutes
prolonged_hypo_54 = data[data['HypoFlag54']].groupby(['Date', 'TimeWindow']).filter(lambda x: len(x) > 3)  #
More than 15 minutes

# Extract information about prolonged hypoglycemia events
prolonged_hypo_70_days_times = prolonged_hypo_70.groupby(['Date',
'TimeWindow']).size().reset_index(name='CountBelow70')
prolonged_hypo_54_days_times = prolonged_hypo_54.groupby(['Date',
'TimeWindow']).size().reset_index(name='CountBelow54')

daily_hypoglycemia_analysis, prolonged_hypo_70_days_times, prolonged_hypo_54_days_times
```

Glycemic Variability

```
# Calculate Coefficient of Variation (CV) for each day
daily_cv = data.groupby('Date').agg(
    MeanGlucose=('CGM', 'mean'),
    StdDevGlucose=('CGM', 'std')
)
daily_cv['CoefficientOfVariation'] = (daily_cv['StdDevGlucose'] / daily_cv['MeanGlucose']) * 100

# Calculate the interquartile range (IQR) for each time window across all days
```

<table>
<tr>
<td></td>
<td></td>
<td>

```
time_window_iqr = data.groupby('TimeWindow').agg(
    Q1=('CGM', lambda x: x.quantile(0.25)),
    Q3=('CGM', lambda x: x.quantile(0.75))
)
time_window_iqr['IQR'] = time_window_iqr['Q3'] - time_window_iqr['Q1']

daily_cv, time_window_iqr
```

</td>
</tr>
<tr>
<td colspan="3"></td>
</tr>
<tr>
<td>Case 10</td>
<td>Data Quality</td>
<td>

```
# Convert the 'Time' column to datetime format
data['Time'] = pd.to_datetime(data['Time'], format='%m/%d/%y %H:%M')

# Extract date from the datetime for counting unique days
data['Date'] = data['Time'].dt.date

# Count unique days
unique_days = data['Date'].nunique()

# Calculate the span of days covered by the dataset
min_date = data['Time'].min().date()
max_date = data['Time'].max().date()
span_days = (max_date - min_date).days + 1  # Including both start and end date

# Assuming readings are taken every 5 minutes, calculate total possible readings in the span
total_possible_readings = span_days * 24 * 60 / 5  # 24 hours, 60 minutes, every 5 minutes

# Calculate the percentage of CGM activity time
actual_readings = len(data)
percentage_activity_time = (actual_readings / total_possible_readings) * 100

# Output summary for quality assessment
unique_days, span_days, percentage_activity_time
```

</td>
</tr>
<tr>
<td></td>
<td>Hyperglycemia</td>
<td>

```
# Define hyperglycemia thresholds
hyper_threshold = 180
severe_hyper_threshold = 250

# Group data by date and calculate percentages of readings above thresholds
daily_hyper_counts = data.groupby('Date')['CGM'].apply(lambda x: (x > hyper_threshold).mean())
daily_severe_hyper_counts = data.groupby('Date')['CGM'].apply(lambda x: (x > severe_hyper_threshold).mean())

# Intraday trends: Break the day into 4-hour time windows
# Define time windows in hours
time_windows = [(0, 4), (4, 8), (8, 12), (12, 16), (16, 20), (20, 24)]
time_window_labels = ["Late night", "Early morning", "Morning", "Afternoon", "Evening", "Night"]

# Function to assign time windows
def assign_time_window(hour):
    for i, (start, end) in enumerate(time_windows):
        if start <= hour < end:
            return time_window_labels[i]
    return "Unknown"

# Assign time window to each reading
data['Time Window'] = data['Time'].dt.hour.apply(assign_time_window)

# Calculate average and maximum glucose in each time window for each day
intraday_avg_glucose = data.groupby(['Date', 'Time Window'])['CGM'].mean().reset_index()
intraday_max_glucose = data.groupby(['Date', 'Time Window'])['CGM'].max().reset_index()

# Merge the average and maximum glucose dataframes for easier analysis
intraday_trends = pd.merge(intraday_avg_glucose, intraday_max_glucose, on=['Date', 'Time Window'],
suffixes=('_Avg', '_Max'))

# Identify days and time windows with severe hyperglycemia (max glucose > 250)
severe_hyper_events = intraday_trends[intraday_trends['CGM_Max'] > severe_hyper_threshold]

# Prolonged hyperglycemia: Find periods of 12 consecutive readings (1 hour) over 250
data_sorted = data.sort_values(by=['Date', 'Time'])
data_sorted['Severe_Hyper_Flag'] = data_sorted['CGM'] > severe_hyper_threshold
data_sorted['Consecutive_Flag'] = data_sorted['Severe_Hyper_Flag'].groupby((data_sorted['Severe_Hyper_Flag']
!= data_sorted['Severe_Hyper_Flag'].shift()).cumsum()).cumcount() + 1

# Find start of periods where there are at least 12 consecutive readings above 250
prolonged_periods = data_sorted[data_sorted['Consecutive_Flag'] == 12]

daily_hyper_counts.describe(), daily_severe_hyper_counts.describe(), severe_hyper_events,
prolonged_periods[['Date', 'Time', 'Consecutive_Flag']].drop_duplicates()
```

</td>
</tr>
</table>

| | | |
|---|---|---|
| | Hypoglycemia | # Define hypoglycemia thresholds<br>hypoglycemia_threshold = 70<br>severe_hypoglycemia_threshold = 54<br><br># Group data by date and calculate percentages of readings below thresholds<br>daily_hypo_counts = data.groupby('Date')['CGM'].apply(lambda x: (x < hypoglycemia_threshold).mean())<br>daily_severe_hypo_counts = data.groupby('Date')['CGM'].apply(lambda x: (x <<br>severe_hypoglycemia_threshold).mean())<br><br># Intraday trends: Analyze hypoglycemia occurrence within the defined time windows<br>intraday_hypo_trends = data[data['CGM'] < hypoglycemia_threshold].groupby(['Date', 'Time<br>Window']).size().reset_index(name='Counts')<br>intraday_severe_hypo_trends = data[data['CGM'] < severe_hypoglycemia_threshold].groupby(['Date', 'Time<br>Window']).size().reset_index(name='Counts')<br><br># Prolonged hypoglycemia: Identify periods below thresholds<br># For hypoglycemia below 70 mg/dL for more than 60 minutes<br>data_sorted['Hypo_Flag'] = data_sorted['CGM'] < hypoglycemia_threshold<br>data_sorted['Consecutive_Hypo_Flag'] = data_sorted['Hypo_Flag'].groupby((data_sorted['Hypo_Flag'] !=<br>data_sorted['Hypo_Flag'].shift()).cumsum()).cumcount() + 1<br><br>prolonged_hypo_periods_70 = data_sorted[(data_sorted['Hypo_Flag'] == True) &<br>(data_sorted['Consecutive_Hypo_Flag'] >= 12)]<br><br># For severe hypoglycemia below 54 mg/dL for more than 15 minutes<br>data_sorted['Severe_Hypo_Flag'] = data_sorted['CGM'] < severe_hypoglycemia_threshold<br>data_sorted['Consecutive_Severe_Hypo_Flag'] =<br>data_sorted['Severe_Hypo_Flag'].groupby((data_sorted['Severe_Hypo_Flag'] !=<br>data_sorted['Severe_Hypo_Flag'].shift()).cumsum()).cumcount() + 1<br><br>prolonged_severe_hypo_periods_54 = data_sorted[(data_sorted['Severe_Hypo_Flag'] == True) &<br>(data_sorted['Consecutive_Severe_Hypo_Flag'] >= 3)]<br><br>daily_hypo_counts.describe(), daily_severe_hypo_counts.describe(), intraday_hypo_trends,<br>intraday_severe_hypo_trends, prolonged_hypo_periods_70[['Date', 'Time',<br>'Consecutive_Hypo_Flag']].drop_duplicates(), prolonged_severe_hypo_periods_54[['Date', 'Time',<br>'Consecutive_Severe_Hypo_Flag']].drop_duplicates() |
| | Glycemic Variability | # Calculate daily coefficient of variation<br>daily_cv = data.groupby('Date')['CGM'].apply(lambda x: (x.std() / x.mean()) * 100)<br><br># Calculate interquartile range for each time window<br>time_window_iqr = data.groupby('Time Window')['CGM'].apply(lambda x: x.quantile(0.75) - x.quantile(0.25))<br><br>daily_cv.describe(), time_window_iqr |

**Table S10: Qualitative Prompts Code written by GPT-4**
*** [ERROR] *** Indicates time when GPT-4 code generation produced error. Subsequent code was then produced.
*** [PAUSE] *** Indicates a time when GPT-4 paused code generation and provides an intermediary text output before proceeding with further code production

# VII. Temperature Variation Experiments

To better understand the performance of the model at different temperature settings, we performed additional experiments varying the temperature for the metric generation tasks. We used ‘gpt-4-turbo-2024-04-09’ and accessed GPT-4 through the OpenAI API (Version 1.54.4) using Python (version 3.9.15). We used the beta assistants feature through the client.beta.assistants.create() function. The code to conduct this experiment and the complete results can be found on the Github repository.

The results are below in Table S11.

| **Metric** | **Accuracy** | | |
|---|---|---|---|
| | **Temperature = 0** | **Temperature = .1** | **Temperature = .5** |
| Number of days the sensor was active | 100% | 100% | 100% |
| % of sensor data captured | 100% | 100% | 100% |
| Mean glucose value | 100% | 100% | 100% |
| Glucose Management Indicator | 100% | 100% | 100% |
| Coefficient of variation | 100% | 100% | 100% |
| Time above 250 mg/dL | 100% | 100% | 100% |
| Time above 180 mg/dL | 100% | 100% | 100% |
| Time between 70 mg/dL and 180 mg/dL | 100% | 100% | 100% |
| Time below 70 mg/dL | 100% | 100% | 100% |
| Time below 54 mg/dL | 100% | 100% | 100% |

**Table S11: Metric generation results: performance at different temperature settings**
